# A multicenter randomized trial for quality of life evaluation by non-invasive intelligent tools during post-curative treatment follow-up for head and neck cancer: Clinical study protocol

**DOI:** 10.3389/fonc.2023.1048593

**Published:** 2023-01-31

**Authors:** Stefano Cavalieri, Claudia Vener, Marissa LeBlanc, Laura Lopez-Perez, Giuseppe Fico, Carlo Resteghini, Dario Monzani, Giulia Marton, Gabriella Pravettoni, Mauricio Moreira-Soares, Despina Elizabeth Filippidou, Aitor Almeida, Aritz Bilbao, Hisham Mehanna, Susanne Singer, Steve Thomas, Luca Lacerenza, Alfonso Manfuso, Chiara Copelli, Franco Mercalli, Arnoldo Frigessi, Elena Martinelli, Lisa Licitra, BD4QoL Consortium, Erlend I. F. Fossen

**Affiliations:** ^1^ Head and Neck Medical Oncology Department, Fondazione Istituto di Ricovero e Cura a Carattere Scientifico Istituto Nazionale dei Tumori, Milan, Italy; ^2^ Department of Oncology and Hemato-Oncology, University of Milan, Milan, Italy; ^3^ Oslo Center for Biostatistics and Epidemiology, University of Oslo, Oslo, Norway; ^4^ Oslo Center for Biostatistics and Epidemiology, Oslo University Hospital, Oslo, Norway; ^5^ Universidad Politécnica de Madrid-Life Supporting Technologies Research Group, ETSIT, Madrid, Spain; ^6^ Applied Research Division for Cognitive and Psychological Science, IEO, European Institute of Oncology IRCCS, Milan, Italy; ^7^ Department of Psychology, Educational Science and Human Movement (SPPEFF), University of Palermo, Palermo, Italy; ^8^ Information Technology Programme Management Office, DOTSOFT, Thessaloniki, Greece; ^9^ DeustoTech, Faculty of Engineering, Universidad de Deusto, Bilbao, Spain; ^10^ Institute of head and neck studies and Education, University of Birmingham, Birmingham, United Kingdom; ^11^ Division of Epidemiology and Health Care Research, JGU - Johannes Gutenberg University, Mainz, Germany; ^12^ Division of Oral and Maxillofacial Surgery - Bristol Dental Hospital, University of Bristol - Bristol Medical School, Bristol, United Kingdom; ^13^ Maxillo-Facial Surgery, Fondazione IRCCS Casa Sollievo della Sofferenza, San Giovanni Rotondo, Italy; ^14^ Maxillo-Facial Surgery, Interdisciplinary Department of Medicine, University of Bari “Aldo Moro”, Bari, Italy; ^15^ MultiMed Engineers srls, Parma, Italy

**Keywords:** mHealth, android, head and neck cancer, QOL, BD4QoL, survivorship, unobtrusive

## Abstract

Patients surviving head and neck cancer (HNC) suffer from high physical, psychological, and socioeconomic burdens. Achieving cancer-free survival with an optimal quality of life (QoL) is the primary goal for HNC patient management. So, maintaining lifelong surveillance is critical. An ambitious goal would be to carry this out through the advanced analysis of environmental, emotional, and behavioral data unobtrusively collected from mobile devices. The aim of this clinical trial is to reduce, with non-invasive tools (i.e., patients’ mobile devices), the proportion of HNC survivors (i.e., having completed their curative treatment from 3 months to 10 years) experiencing a clinically relevant reduction in QoL during follow-up. The Big Data for Quality of Life (BD4QoL) study is an international, multicenter, randomized (2:1), open-label trial. The primary endpoint is a clinically relevant global health-related EORTC QLQ-C30 QoL deterioration (decrease ≥10 points) at any point during 24 months post-treatment follow-up. The target sample size is 420 patients. Patients will be randomized to be followed up using the BD4QoL platform or per standard clinical practice. The BD4QoL platform includes a set of services to allow patients monitoring and empowerment through two main tools: a mobile application installed on participants’ smartphones, that includes a chatbot for e-coaching, and the Point of Care dashboard, to let the investigators manage patients data. In both arms, participants will be asked to complete QoL questionnaires at study entry and once every 6 months, and will undergo post-treatment follow up as per clinical practice. Patients randomized to the intervention arm (n=280) will receive access to the BD4QoL platform, those in the control arm (n=140) will not. Eligibility criteria include completing curative treatments for non-metastatic HNC and the use of an Android-based smartphone. Patients undergoing active treatments or with synchronous cancers are excluded.

Clinical Trial Registration: ClinicalTrials.gov, identifier (NCT05315570).

## 1 Introduction

Depending on disease stage, head and neck cancer (HNC) can be cured either with single modality or with multimodal treatments, consisting of various combinations of surgery, radiotherapy and chemotherapy. Despite treatment with curative intent, loco-regional recurrences and/or distant relapses are frequent. Moreover, these therapeutic approaches result in significant acute toxicities and late sequelae. Therefore, quality of life (QoL) is often impaired in these survivors. It is known that QoL is a prognostic factor because it is related to overall survival in cancer patients (1) and to loco-regional control in HNC patients (2). Published studies suggest that even though people having undergone treatment for HNC usually recover their global QoL by 12 months after treatment, persistent late sequelae are observed, notably poor physical functioning, fatigue, xerostomia and sticky saliva (3).

Three main validated QoL questionnaires will be used: EORTC QLQ-C30 for all types of malignancies (4), EORTC HN43 [an updated version of the formerly used H&N35 questionnaire (5)] for HNC patients only (6), EQ-5D-5L (7) which is a patient-reported measurement focused on five domains (mobility, self-care, usual activities, pain/discomfort, and anxiety/depression), that can be used for assessment of health status and also for health technology assessment (HTA) (8). Interestingly, in some subsets like nasopharyngeal carcinoma, differences in QoL are independent predictors of prognosis (9). In the context of patient-reported outcome measurements (PROMs), the cancer behavior inventory (CBI) (10) and its brief version (CBI-B) (11) are validated instruments aimed at measuring self-efficacy strategies for coping with cancer. Specifically, self-efficacy for coping with cancer is a psychosocial construct referring to people’s beliefs about their capabilities of effectively executing behaviors that occur in the course of dealing with a cancer diagnosis, cancer treatments, and transitioning to survivorship (12). The results of a recent meta-analysis attest that cancer patients with higher coping self-efficacy report a higher quality of life (12). In the clinical and research contexts, the CBI-B represents a useful adjunct to other PROMs because it allows detection of cognitive changes in personal, control, and psychosocial adjustment and identification of patients who need further psychosocial services (11).

In order to minimize the risk of data misinterpretation and to maximize the precision and the accuracy in measuring QoL variations effectively, clinically meaningful differences in QoL scales have been suggested. In particular, when considering an overall indicator such as global health status according to EORTC QLQ-C30 a deterioration is considered clinically relevant if there is a reduction in at least 10 points of the score (13, 14).

Predictive factors for identifying which HNC patients are at higher risk of suffering long-term poor QoL have not yet been identified. To date, the only way we can work out if someone has poor long-term QoL is to administer repeated QoL questionnaires during follow-up. Avoiding QoL deterioration, even if temporary, is a critical clinical need. An ambitious goal would be to detect early variations in QoL (or in other measures potentially predicting later QoL deterioration) that translate into a tangible benefit (e.g., early diagnosis of incoming and evolving health status) that improves long-term QoL in HNC survivors. The combination of big-data analysis techniques and innovative use of information technology (IT) tools (e.g., smartphones) may allow targeted interventions to improve QoL. The results of a pilot study conducted on patients treated with palliative intent for gynecological cancers illustrated the potential benefits of such an approach (15). A mobile health intervention collecting both PROMs and activity data as a measure of health status was shown to be feasible and acceptable. Moreover, this was perceived to be effective in improving symptom management in patients with advanced gynecologic cancers. Most of the evidence on eHealth solutions in HNC patients is made of pilot or feasibility studies. The results of a non-blinded randomized controlled trial evaluating Oncokompas, an eHealth application aimed at supporting self-management of symptoms and health-related quality of life in cancer survivors, including HNC, were published in 2020 (16). Oncokompas did not improve knowledge, skills, and confidence in self-management in cancer survivors. However, the median follow-up of patients included in this study was only 6 months (after treatment conclusion), thus possibly preventing the observation of late QoL declines in most subjects. The study included 185 HNC patients (99 in the intervention arm, 86 in control one) enrolled across 14 Dutch hospitals in 19 months. This means an average of 9 patients recruited per year per center. Only 68 out of the 99 (68%) HNC patients in the intervention arm, and only 64 out of the 86 patients (74%) in the control arm returned a second questionnaire after 6 months. Therefore, only 132 of the 185 HNC patients (71%) remained in the study. This means that a 30% drop-out was observed over a six-month period.

The main aim of the present study is to anticipate and reduce, with the use of the non-invasive Big Data for Quality of Life (BD4QoL) platform (described below), the proportion of HNC survivors experiencing a clinically relevant reduction in QoL. If the participants report or are identified as having a significant finding during monitoring, a specific set of interventions will be applied if symptoms and findings on monitoring indicate their application. This clinical trial was designed and set up in the framework of the BD4QoL research project (full project title “Big Data Models and Intelligent tools for Quality of Life monitoring and participatory empowerment of head and neck cancer survivors”), that lasts 5 years and is funded by the European Commission (further details are specified in Section 7) (17). The BD4QoL Consortium is an interdisciplinary partnership made of several partners from seven European Countries, combining the complementary competences, skills, structural and infrastructural capabilities.

The adoption of mobile technologies of everyday use (i.e., embedded into standard mobile phones) for behavior reconstruction and linkage of behavior modifications to quality of life indicators, and the realization of predictive models for quality of life modifications will allow seamless and unobtrusive data capture over time, making the execution of clinical investigations more precise and less burdensome as compared to standard (manual) data capture. Artificial intelligence (AI) algorithms, including Machine Learning, Transfer Learning, Deep Learning-Based Models, Knowledge-Based Activity Models (Expert Activity Models – EAM) for behavior patterns recognition, IBM Watson technologies for affective computing such as Tone Analyzer, Natural Language Classifier, Natural Language Understanding, will be used.

## 2 Methods and analysis

### 2.1 Study objectives and endpoints

The overall aim of this study is to assess if QoL deterioration can be anticipated and prevented by the addition of the BD4QoL platform to standard of care (SoC) versus SoC alone, in HNC survivors post-treatment with up to 24-month follow-up. Primary and secondary objectives and endpoints are specified in **Table 1**, exploratory ones in **Table 2**.

**Table 1:** Study objectives and endpoints.

	Objective	Endpoint
***Primary***	To reduce the proportion of HNC subjects experiencing a clinically meaningful deterioration of QoL between at least 2 visits during post-treatment follow-up.	The proportion of HNC survivors experiencing a clinically relevant deterioration in the global health scale of the EORTC QLQ-C30 [decrease ≥10 points, as defined in (13,14)] within the study observation (up to 24 months) period during post-treatment follow-up.
***Secondary***	To delay the time to the first clinically meaningful deterioration of QoL between at least 2 visits during post-treatment follow-up.	The time to first clinically relevant deterioration of EORTC QLQ-C30 global score [decrease ≥10 points, as defined in(13,14)] measured within the study observation (up to 24 months) period during post-treatment follow-up.
	To reduce the proportion of HNC subjects experiencing a clinically relevant deterioration in pre-specified QoL domains between at least 2 visits during post-treatment follow-up.	The proportion of HNC survivors experiencing a clinically meaningful deterioration [as defined in (13,14)] of pre-specified EORTC QLQ-C30 scales (emotional functioning, role functioning, sleep) within the study observation (up to 24 months) period during post-treatment follow-up.
	To reduce the proportion of HNC subjects experiencing a clinically meaningful deterioration in pre-specified head and neck cancer specific QoL domains between at least 2 visits during post-treatment follow-up.	The proportion of HNC survivors experiencing a clinically relevant deterioration of pre-specified EORTC QLQ-HN43 scales (swallowing, problems with teeth, problems opening mouth, speech, social eating, fear of progression, emotional functioning, fatigue) within the study observation (up to 24 months) period during post-treatment follow-up.
	To reduce the proportion of HNC subjects experiencing a clinically meaningful deterioration in health status between at least 2 visits during post-treatment follow-up.	The proportion of HNC survivors experiencing a clinically meaningful deterioration [as defined in (7)] of EQ-5D-5L domains (mobility, self-care, usual activities, pain/discomfort, anxiety/depression) within the study observation (up to 24 months) period during post-treatment follow-up

**Table 2:** Exploratory objectives and endpoints.

Exploratory objective	Exploratory endpoint
To assess the association of clinically relevant variations in QoL [EORTC QLQ-C30 and HN43 global scores (13, 14)] with disease recurrence and survival in HNC survivors within the study observation (up to 24 months) period during post-treatment follow-up.	Association of disease-free survival (DFS), event-free survival (EFS) and overall survival (OS) with clinically relevant variations (either increase or decrease, as appropriate according to scales) of EORTC QLQ-C30 and HN43 questionnaires, as defined in (13, 14).
To To analyze time-dependent variations of QoL (EORTC QLQ-C30 and HN43 scores) within the study observation (up to 24 months) period during post-treatment follow-up in HNC survivors assessing their association with recurrence and survival.	Association of disease-free survival (DFS), event-free survival (EFS) and overall survival (OS) with time-dependent clinically relevant variations (either increase or decrease, as appropriate according to scales) of EORTC QLQ-C30 and HN43 questionnaires, as defined in (13, 14).
To To analyze QoL scores (EORTC QLQ-C30 and HN43 scores) within the study observation (up to 24 months) period during post-treatment follow-up in nasopharyngeal cancer patients assessing their association with recurrence and survival.	Association of disease-free survival (DFS), event-free survival (EFS) and overall survival (OS) with clinically relevant variations (either increase or decrease, as appropriate according to scales) of EORTC QLQ-C30 and HN43 questionnaires, as defined in (13, 14), in nasopharyngeal carcinoma patients.
To reduce the proportion of HNC subjects experiencing a clinically meaningful deterioration in pre-specified EORTC QLQ-HN43 scales (not included in secondary endpoints) between at least 2 visits during post-treatment follow-up.	The proportion of HNC survivors experiencing a clinically meaningful deterioration [as defined in (13, 14)] of pre-specified EORTC QLQ-HN43 scales (pain in the mouth, problems with senses, body image, dry mouth and sticky saliva, coughing, social contact, neurological problems, sexuality, problems with shoulder, skin problems) within the study observation (up to 24 months) period during post-treatment follow-up.
For HNC survivors randomized in the BD4QoL platform group, using artificial intelligence techniques to build models to predict a QoL deterioration [EORTC QLQ-C30 and HN43 scores, EQ-5D-5L as defined in (7, 13, 14)] between at least 2 visits during within the study observation (up to 24 months) period during post-treatment follow-up.	The association of all the health-related data recorded by the BD4QoL platform **Supplementary Material** registered continuously within the study observation (up to 24 months) period during post-treatment follow-up with clinically relevant variations (either increase or decrease, as appropriate according to scales) of EORTC QLQ-C30 and HN43 questionnaires, as defined in (7, 13, 14)
To assess the association of clinically relevant variations of QoL [EORTC QLQ-C30 and HN43 global scores (13, 14)] with self-efficacy for coping with cancer in HNC survivors within the study observation (up to 24 months) period during post-treatment follow-up.	Association of clinically relevant variations (either increase or decrease, as appropriate according to scales) of EORTC QLQ-C30 and HN43 questionnaires, as defined in (13, 14), and self-efficacy for coping with cancer, defined as mean of the item scores at the CBI-B (11).
To reduce the proportion of HNC subjects experiencing a clinically meaningful deterioration of QoL between at least 2 visits during post-treatment follow-up, stratifying according to patients completing treatment within 12 months versus after 12 months.	The proportion of HNC survivors experiencing a clinically meaningful global health-related EORTC QLQ-C30 QoL deterioration [decrease ≥10 points, as defined in (13, 14)] within the study observation (up to 24 months) period during post-treatment follow-up. Stratification according to timing after study completion (less than 12 months versus more than 12 months)
To assess the economic impact on HNC survivor care and the viability, usability, and trust of using the BD4QoL platform (Health Technology Assessment, HTA).	Incremental Cost-Effectiveness Ratio measured in €/QALY. Measures of viability, usability, and trust.

### 2.2 Study design

This is a multicenter, international, two-arm, randomized (2:1 ratio), open-label, superiority trial, designed to evaluate the proportion of HNC survivors experiencing a clinically meaningful QoL deterioration [reduction of at least 10 points in EORTC QLQ-C30 global health status (13, 14)] between at least 2 visits during post-treatment follow-up (up to 24 months from randomization) with the use of the BD4QoL platform through the web-forms tool for QoL questionnaires answers and through continuous monitoring by a mobile application (App) installed on the study subject’s mobile phone in comparison to those without the BD4QoL platform (SoC). If a clinically relevant deterioration in global health, measured with the EORTC QLQ-C30 global health scale [decrease ≥10 points, as defined in (13, 14)] is detected during the study period, the subject participation in the study will be interrupted (because the aforementioned decrease would imply that the primary endpoint is not met for a specific participant).

Patients will be followed up as per clinical practice (18, 19):

a. All subjects will be asked to complete validated questionnaires [EORTC QLQ-C30 (4), QLQ-HN43 (6), and EQ-5D-5L (7), CBI-B (11)] and PREM on up to 5 occasions over a 2-year period, at randomization (0-month of follow-up), then once every 6 months ± 2 months (after 6, 12, 18, 24 months from randomization).

b. Participants in both arms will be offered routine SoC and asked to complete validated QoL questionnaires at regular intervals (described above). In addition, those in the intervention arm will be offered the device-generated data collection platform (BD4QoL device *plus* QoL validated questionnaires *plus* counseling and physician contact triggered by the data generated by the platform [early intervention] *plus* a possible psychological effect of the whole activities in the interventional arm). A 2:1 randomization will be used (2/3 of subjects with BD4QoL platform versus 1/3 of subjects without). Participants randomized to the control arm will not receive the above-mentioned platform; they will fill in the same validated questionnaires on web-based instruments and will be followed up as per usual clinical practice.

**Figure 1:**
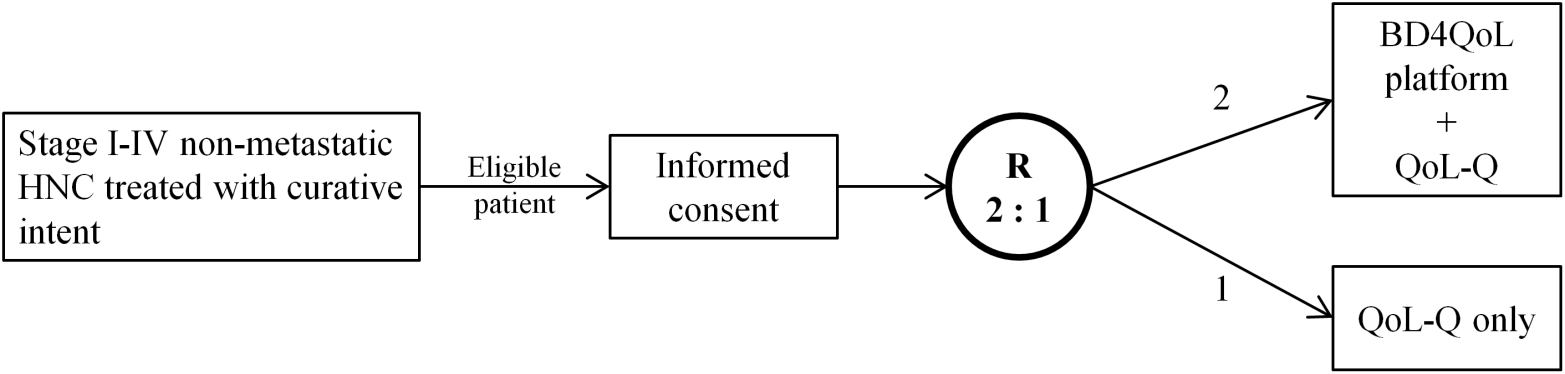
Study design of the randomized controlled trial.

The study design is summarized in **Figure 1**. The choice of unequal randomization in randomized controlled trials is rarely resorted to. The main reasons for its justification have been reviewed by Dibao-Dina et al. (20): obtaining more safety data, exposing fewer patients to the potentially inferior group, increasing patients acceptability, reducing costs.

In this scenario, the 2:1 ratio was chosen for the following reasons: i) the study devices are deemed to be safe and able to provide timely monitoring of participants health status, thus 1) possibly improving their condition as compared to the control group and 2) minimizing the number of patients not exposed to this potential benefit; ii) the platform acts unobtrusively, and it favors contact with healthcare professional which is normally very well accepted and pursued by patients; moreover it is designed to allow for patients’ empowerment; iii) we anticipate that costs associated with SoC not supported with the BD4QoL platform will be superior because subjects randomized in the intervention arm are deemed to experience less adverse events than controls. In this context, a cost-effectiveness analysis is planned as an exploratory endpoint (**Table 2**).

As stated by Palmer et al., favoring the experimental therapy is warranted in trials of potentially significant public health benefit (21). Unequal allocation schemes can improve recruitment and partially satisfy the individual ethics criterion (the study participants have a higher probability of being randomized in the intervention arm than in control one). They can also be useful if widespread knowledge about the control therapy already exists and if more understanding is desired about the new treatment.

The randomization procedure will be web-based, centralized, computerized (software-generated randomization list using RedCap web-based platform) (22, 23). Local research teams will be blinded to the randomization list (local people designated to the randomization procedure will not know the randomization list, but clinical investigators will not be blinded to the outcome of the randomization). The biostatistician doing the analysis of the trial will be blinded to the list as well until the statistical analysis plan, previously defined, is signed.

The EORTC QLQ-C30 (4), EORTC QLQ-HN43 (6), EQ-5D-5L (8), and CBI-B (11) evaluations will be performed at 0-, 6-, 12-, 18-, 24-month from randomization. The time zero is considered to be the date of randomization. The scores of the EORTC QLQ instruments [as defined in (13, 14)] will be compared to the respective scores of the previous study time-point.

### 2.3 Study population

Each participating Center (recruiting patients in Italy and the United Kingdom) will enroll consecutive patients according to the eligibility criteria listed below.

#### 2.3.1 Inclusion criteria

1. Effectively cured histologically defined head and neck squamous cell carcinoma (HNSCC) from one of these subsites: oral cavity, nasopharynx, hypopharynx, larynx, Human Papillomavirus (HPV)-positive or negative oropharynx, nasal cavity, paranasal sinuses. Non-metastatic salivary gland cancer (SGC) of any histological type can be included only if curative or postoperative radiotherapy included the neck:
  - For p16-negative or p16-unknown HNSCC (including nasal cavity and paranasal sinuses), stage I, II, III, IVa or IVb (no IVc) according to UICC/AJCC 8^th^ edition. Regional neck metastases from squamous cell carcinoma from unknown primary head and neck sites are allowed.
  - For nasopharyngeal cancer (NPC), stage I, II, III, IVa (no IVb) according to UICC/AJCC 8^th^ edition. Regional neck metastases from EBV-positive carcinoma from unknown primary head and neck sites are allowed.
  - For SGCs, stage III, IVa or IVb according to UICC/AJCC 8^th^ edition treated with radiotherapy that included the neck (either post-operative radiation or radical treatment in case of unresectable disease).
  - For p16-positive oropharyngeal squamous cell carcinoma, stage I, II or III according to UICC/AJCC 8^th^ edition. Regional neck metastases from p16-positive and/or HPV-positive squamous cell carcinoma from unknown primary head and neck sites are allowed.
2. Patients having completed treatment with curative intent (including any single modality or multimodal approach) within 10 years at the time of accrual.
3. Patients being disease-free at the time of accrual. Patients will be deemed in complete remission if the clinical examination is negative for recurrence; clinical examination should be preferably, but not mandatorily, integrated with unequivocal radiological imaging that shows the absence of disease (in case of doubt, further radiological imaging should be performed or integrated with cyto/histological samples of the area with suspected disease persistence and the exams will have to be consistently negative) after at least three months following treatment completion.
4. Ability to fill in questionnaires as per protocol.
5. Geographical accessibility and willingness to be followed-up for up to 2 years with information-technology (IT) devices in addition to questionnaires.
6. Age ≥ 18 years.
7. Signed informed consent.
8. Willingness to use their smartphone and their Internet access for the study.
9. Smartphone having the following minimum characteristics:
  1. RAM: Minimum of 2 GB
  2. Storage: Minimum of 512 MB free storage
  3. Operating system: Android version 7 (Nougat) or upper.

#### 2.3.2 Exclusion criteria

1. Distant metastases (the following populations are excluded: stage IVc HPV-negative HNSCC and SGC, stage IV p16-positive oropharyngeal squamous cell carcinoma, stage IVb NPC).
2. Thyroid cancers, non-melanoma skin cancers (e.g. squamous cell carcinoma of the skin, skin basal cell carcinoma, skin adnexal carcinoma), and non-carcinoma HNC (e.g. melanoma, sarcoma, etc.) are excluded.
3. Subjects with previous malignancies (except localized non-melanoma skin cancers, and the following *in situ* cancers: bladder, gastric, colon, esophageal, endometrial, cervical/dysplasia, melanoma, or breast) unless a complete remission was achieved at least 5 years prior to study entry AND no additional therapy is required during the study period. Premalignant lesions (e.g., leukoplakia, erythroplakia, lichen etc.) are allowed.
4. Participation in clinical trials with other experimental agents within 30 days of study entry or concomitant treatment with experimental drugs.
5. Patients unable to comply with the protocol, in the opinion of the investigator.
6. Any known or underlying medical conditions that, in the opinion of the investigator, could adversely affect the ability of the participating subject to comply with the study.
7. Having a smartphone operating system other than Android.

### 2.4 Instruments

#### 2.4.1 Questionnaires

The EORTC QLQ-C30 is composed of both multi-item scales, including functional scales, symptom scales, a global health status/QoL scale, and single-item measures. Each of the multi-item scales includes a different set of items. Each item is represented once in each scale, meaning that no item occurs in more than one scale (24).

All scales and single-item measures range in score from 0 to 100. A high scale score represents a higher response level. Thus, a high score for a functional scale represents a high/healthy level of functioning, a high score for the global health status/QoL represents a high QoL, but a high score for a symptom scale/item represents a high level of symptoms/problems.

The principle for scales scoring is the same:

1. Estimate the average of the items that contribute to the scale (raw score).
2. Use a linear transformation to standardize the raw score (ranging from 0 to 100); a higher score in the function scales represents a higher (“better”) level of functioning, or a higher score in the symptom scales a higher (“worse”) level of symptoms.

The EORTC QLQ-HN43 (an update of QLQ-H&N35) is meant for use in head and neck cancer patient populations varying in disease stage and treatment modality (i.e. surgery, chemotherapy and radiotherapy). It should always be complemented by the EORTC QLQ-C30. As described for EORTC QLQ-C30, all scales and single-item measures of EORTC QLQ-HN43 range in score from 0 to 100.

The EQ-5D-5L is a standardized measure of health status developed by the EuroQol Group in order to provide a simple, generic measure of health for clinical and economic appraisal (25).

The EQ-5D-5L consists of the EQ-5D-5L descriptive system and the EQ Visual Analogue Scale (EQ VAS). The descriptive system comprises five dimensions (mobility, self-care, usual activities, pain/discomfort, anxiety/depression). Each dimension has five levels: no problems, slight problems, moderate problems, severe problems, and extreme problems. The respondent is asked to indicate his/her health state by ticking (or placing a cross) in the box against the most appropriate statement in each of the five dimensions. This decision results in a 1-digit number expressing the level selected for that dimension. The digits for 5 dimensions can be combined in a 5-digit number describing the respondent’s health state. It should be noted that the numerals 1-5 have no arithmetic properties and should not be used as an ordinal score. The EQ VAS records the respondent’s self-rated health on a 20 cm vertical visual analog scale with endpoints labeled ‘the best health you can imagine’ and ‘the worst health you can imagine’. This information can be used as a quantitative measure of health as judged by the individual respondents (26).

The Cancer Behavior Inventory - Brief (CBI-B) was developed as a measure of self-efficacy strategies for coping with cancer, based on self-regulation and self-efficacy theories (11). It consists of 12 items (rated 1 = not at all confident to 7 = totally confident) and was derived from the longer version of the Cancer Behavior Inventory (CBI) (10, 27). The CBI-B represents a comprehensive and valid brief measure of self-efficacy for coping with cancer that could be easily used as a PROM. It has a consistent factor structure across several types of cancer and established good psychometric qualities (28). The CBI-B total score can be computed by averaging single item scores through arithmetic mean and, thus, ranges in value from 1 to 7.

#### 2.4.2 BD4QoL platform

In the BD4QoL project, the opportunities linked to mobile-health (mHealth) and digital phenotyping (29) will be used to continuously monitor QoL trajectories in HNC patients and to detect early related events that need further attention from patients, clinicians, or both.

The BD4QoL platform consists of a set of services to allow patient monitoring and empowerment through two main tools: a Point of Care (PoC) web application to manage all patients’ data and follow-up by clinical investigators, and a mobile application (App) installed on participating subject’s smartphone. Also, a web-form tool is delivered to allow the QoL questionnaire completion.

A preliminary feasibility and users acceptability assessment has been performed at the Istituto Nazionale dei Tumori in Milan, Italy (unpublished, data not shown). This preliminary survey has outlined the preferences of users in terms of mobile technologies to be adopted (mobile phones preferred as compared to home and wearable sensors/devices). Moreover, a pilot study was performed on healthy volunteers to measure the accuracy, precision, and uniformity of data collected by the different mobile phones used by the study participants. The acceptability and usability of the platform were assessed as well within the same preliminary pilot study (data not shown).

To achieve the study objectives, the BD4QoL platform will collect the following data which will be used to identify behavioral and affective traits associated with study outcomes.

Sensors in mobile phones will provide the following readings (further details in **Supplementary Material**):

- Accelerometer (x,y,z measurements).
- Global Positioning System - GPS (Lat, Long).
- Ambient light (measurement of light in the room/area where the mobile device is located).
- Screen (Status ON or OFF for smartphone device screen).
- Activity (type of activity with which the person engages, which can be one of these: Still, Walking, Running, On_Bicycle, In_Vehicle)
- Daily connections to wifi networks (naming of wifi connections as well as corresponding duration).

The following data are detected from the mobile device’s operating system:

- Phone usage logs (total count and timestamps of three types of events – incoming, outgoing and missed calls – with encrypted [that is hashed] collection of associated personal identifiable information, as well as total count and timestamps of incoming and outgoing/replied text messages).
- Phone applications usage (identification and seconds or minutes of total and detailed usage of any smartphone applications the study participant is using; for certain specific social media network or communication mobile apps, more specific information, that is as duration of app usage per day, is collected based on participant’s permissions; these social applications include Facebook, Messenger, Whatsapp, Telegram, Viber, Zoom, Instagram; no information about the people with whom communication is made nor the content of the communication is collected).

The following data are collected from external datasets:

- Steps (daily and per hour number of steps completed through connection to external dataset from Google Fit cloud).
- Identification of places (Points of Interest) visited, based on participant’s permissions, through the correlation of one’s GPS signal (per day and minute) with external datasets from Foursquare and OpenStreet maps.

The above data will be used to infer activities and behaviors which have a high likelihood of being meaningfully related to participants’ QoL trajectories. A list of such activities and behaviors, with an indication of the technical methods to be used for reconstruction as well as an assessment of reliability, is presented in **Supplementary Material**.

**Figure 2:**
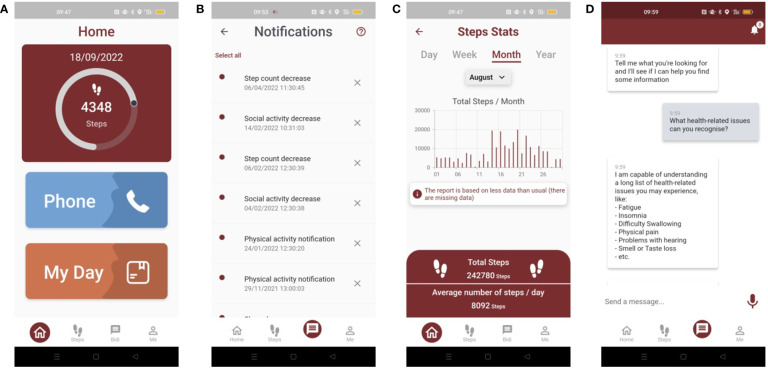
Screenshot of the BD4QoL study mobile app: **(A)** homepage, **(B)** notification dashboard, **(C)** monthly statistics of step count, **(D)** example of BiDi chatbot conversation.

The BD4QoL App, available for Android (**Figure 2**), as listed above, will be able to collect and store information about the following domains: mobility, physical activity, activities of daily living, instrumental activities of daily living, socialization, cognitive function, health-related activities as well as personal affective data (further details in **Supplementary Material**). A summary of the findings and the supporting data will be available to the patient and clinical investigators (e.g., physicians, nurses), through a dashboard available on mobile devices for patients and through the PoC web application for clinical investigators. The data collected by the mobile App will not be available to the technology manufacturer and will be transferred in quasi-real-time (i.e., as soon as a connection for data transfer is available) to the central BD4QoL repository.

In the interval between visits, study participants, allocated in the intervention arm, will be able to interact electronically with a chatbot(implementation based on IBM Watson technology), which will be part of the BD4QoL App. The chatbot is an application based on a conversational user interface (30) to empower patients to manage their QoL and health under the supervision of clinical investigators. The chatbot will have a series of electronic coaching (e-coaching) functions that include: (i) dialogue management that allows the patient to be counseled by chatting electronically in a structured and effective way; (ii) management of two-way communications with healthcare professionals [e.g., for the patient to request specific support in case of special needs, or for the chatbot to invite the patient for a visit in case of an early detection of health-related QoL (HRQoL) deterioration or health issues; identified people will have to be listed on the delegation log by the Principal Investigator (PI)]; (iii) detection of affective traits embodied in the e-coach/patient dialogue, through sentiment analysis and emotion analysis technologies to infer information about the participant mood (details about data privacy are reported in the specific section of this protocol). The latter element can be used to both re-adapt the chatbot counseling strategy as well as to provide additional information on the subject’s mood to clinical investigators. The adverse events that the chatbot will be able to recognize will be the following: fatigue, malaise, fever, excessive sleepiness, difficulty sleeping, depression, change in social circumstances, neck swelling, facial pain, difficulty breathing, nose bleeds, difficulty speaking, dry mouth, tooth loss, muscle weakness, ear pain, difficulty hearing, tinnitus, vertigo, nausea, diarrhea, constipation, difficulty seeing, dry eye, eye pain, nervous eyelid, eye floaters, swollen eye, bleeding eyes, eye-watering, sexuality issues, weight loss, difficulty swallowing, mouth sores, appetite loss, difficulty opening mouth, difficulty eating, increased sensitivity to smells, no taste. Further details about adverse events and potential chatbot responses are reported in **Supplementary Table 2**.

The platform will provide the investigators with real-time data on device usage (e.g., number and type of alerts and chatbot interactions by pts), and it will integrate the electronic case report forms (eCRF) as a study monitoring dashboard through the PoC web application.

The BD4QoL platform used in this trial is not to be considered a medical device and is used for experimental assessment only. No drugs will be suggested by the automated chatbot responses. Tips provided by the chatbot regarding detected symptoms are also not to be considered clinical advice by any means and should not be a substitute for conversations with a member of trained medical personnel. A relevant disclaimer in this sense is also clearly shown by the chatbot itself to the study participant.

### 2.5 Sample size calculation and statistical plan

#### 2.5.1 Sample size

The primary endpoint is the proportion of HNC survivors experiencing a clinically relevant deterioration in global health-related QoL as previously defined.

In the European BD2Decide project (31), funded by the Horizon 2020 program and concluded in November 2019, the analysis of QoL questionnaires was performed on over 450 stage III-IV HNSCC patients (recruited from Italy, Germany, and the Netherlands; data cut-off date 23^rd^ September 2019): at least two consecutive EORTC QLQ-C30 questionnaires filled in after at least 6 months of follow-up were available for 117 patients; among them, a clinically meaningful deterioration of QoL (reduction of at least 10 points) was observed in 22 (19%) cases during post-curative treatment follow-up.

The second historical cohort comprised 65 patients from Mainz (Germany) having EORTC QLQ-C30 questionnaires completed 24 and 36 months after curative surgery (32). In this population, the percentage of subjects experiencing a deterioration ≥ 10 points in EORTC QLQ-C30 global health status from the 24^th^ to the 36^th^ months after treatments was 23%.

In the Head and Neck 5000 project (UK) (33) repeated global health scores at 12 and 36 months after treatment were available for 1241 people with HNC. A clinically meaningful deterioration of QoL (reduction of at least 10 points) was observed in 18% of these subjects.

In these three existing studies, the average percentage (arithmetic mean) of HNC patients experiencing a clinically meaningful deterioration of EORTC QLQ-C30 global score (reduction of at least 10 points) during follow-up was 20% (19% BD2Decide, 23% Mainz, 18% HN5000). These findings are preliminary and will be explored further as part of the European Union (EU) program supporting this study.

The primary endpoint of this study is the proportion of patients showing a reduction of at least 10 points in EORTC QLQ-C30 global health status score in HNC survivors. Therefore, considering the null hypothesis (*H_0_*) of no difference for the primary endpoint, the proportion of HNC survivors showing a reduction of at least 10 points in EORTC QLQ-C30 global health status during 24-month FU in the control group is 20% (*µ_0_*), as derived from the historical benchmark data.

Considering the alternative hypothesis (*H_1_*), the desired proportion of HNC survivors showing a reduction of at least 10 points in EORTC QLQ-C30 global health status during FU in the intervention group is 10% (*µ_1_*).

The minimal clinically relevant difference (*δ*) [superiority randomized controlled trials (RCTs)] between the null hypothesis (*H_0_*) of no difference and the alternative hypothesis (*H_1_*) is 10%. We fixed the significance level (*α* = 0.05, one-tailed) and the power (*1-β* = 0.80, at least). It has been calculated a group sample sizes of 112 subjects (the control group) and 224 subjects (the intervention group) will result in 80% power (*1-β*) to detect a difference of 10% between the null hypothesis (*H_0_*) that both group proportions are 20% and the alternative hypothesis (*H_1_*) that the proportion in group under study is 10%, using a one-sided chi-squared test (*χ* ^2^ test) (Mantel-Haenszel test) and with a significance level of 0.05 (*α*, one-tailed) (PASS Sample Size 2020 statistical software).

With a supposed drop-out of 20%, it has been calculated a group sample size of 140 subjects (the control group) and 280 subjects (the intervention group), for a total cohort of 420 participants.

The data will be published in clinicaltrials.gov and will be reported to study PIs and to the Funding Authority. The protocol writing committee will have the final decision regarding study termination.

#### 2.5.2 Evaluable cases for primary endpoint

For the purposes of analysis, the study populations are defined in **Table 3**.

**Table 3:** Populations for analysis.

Population	Description
All screened	All participants who sign the informed consent form.
Intent-to-treat (ITT)	All randomized participants whether or not the randomized intervention was administered. This population will be based on the study intervention to which the participant was randomized and will be the primary population for the analysis of efficacy data.
Per-protocol	All randomized participants completing at least two QoL questionnaires 6 months apart, as long as the study participant is disease-free. Participants will be analyzed according to the intervention they actually received.

#### 2.5.3 Questionnaires (both intervention and control arms)

For the primary endpoint, a randomized patient will be considered evaluable given that at least two QoL questionnaires have been completed with the following timing: timing between 2 questionnaires ≥ 6 months as long as the study participant is disease-free.

#### 2.5.4 BD4QoL platform (intervention arm only)

The primary endpoint will be analyzed based on intention to treat. In addition to the criteria described in point 6.2.1, a study subject randomized in the intervention arm will be considered for the per-protocol analysis, if the BD4QoL platform is turned on for at least 70% of daily hours (non-sleep activity, as defined in **Supplementary Table 1**).

#### 2.5.5 Data collection

At study entry, the following data will be collected and recorded in the electronic case report form (eCRF): date of signed informed consent, gender, marital status, education, date of primary tumor diagnosis, comorbidity (ACE27), baseline body mass index (BMI), age at primary tumor diagnosis, primary tumor site (International Classification of Diseases for Oncology, ICD-O code), HPV and/or p16 status (mandatory if primary tumor site = oropharynx), serum plasma Epstein-Barr Virus (EBV)-DNA (optional if primary tumor site = nasopharynx), EBER (EBV Encoded small RNAs) on tumor specimen (optional if primary tumor site = nasopharynx), Baseline (pre-treatment) cTNM staging^19^ (AJCC/UICC VIII edition), details about surgery, radiotherapy, systemic therapy, date of treatment completion.

At each follow-up, the following data will be collected and recorded in the eCRF: date of consultation, timing after treatment completion (first follow-up; +6 months; +12 months; +18 months; +24 months; other), BMI, disease status (recurrence; date of recurrence; site of recurrence: loco-regional and/or distant and/or second primary tumor), new non-cancer-related medical events [Medical Dictionary for Regulatory Activities (MedDRA)], grade of non-cancer-related medical events according to CTCAE (Common Terminology Criteria for Adverse Events) version 5.0, date of onset and resolution of non-cancer-related medical events, intervention for non-cancer-related medical events (no intervention; surgery; rehabilitation; medical intervention, if yes, to be specified according to Anatomical Therapeutic Chemical Classification System [ATC]; Psychological intervention; other).

#### 2.5.6 Statistical plan

A comprehensive statistical plan will be prepared, discussed, and consented with the study team after data collection is completed and before the data analysis starts. The current plan is as follows: descriptive statistics will be generated for all clinical characteristics and outcome measures as appropriate (for continuous variables: sample size [n], mean, standard deviation, median, minimum, maximum, kurtosis, skewness; for categorical variables: frequency, percentage). All the analyzes will be performed using the ITT population and per-protocol. Missing data will be considered as lack of data. An analysis of missingness will be performed to understand whether there is relevant selection bias and appropriate methods to handle missing data will be applied according to the plausibility of the missingness mechanisms in the data.

The full sample size is expected to be accrued in an 18-month period. An interim analysis (only for checking patient number) will be performed after 12 months from the study start. If at least 50% of the planned population (≥210 subjects) have been randomized within the first 12 months of recruitment, the trial will continue with no modifications. Otherwise (randomized patients ≤ 209 patients in the first 12 months of study conduction), the BD4QoL Consortium will discuss, plan and agree strategies to foster patient recruitment, and eventually ask the funding Institution for a project extension. In case of any change in the clinical study protocol, a major amendment to the protocol will be submitted to the Ethical Committees of the participating sites.

All the analyzes, summaries, listings will be carried out using SAS statistical software (SAS version 9.4 of the SAS System, SAS Institute Inc., Cary, NC, USA). All statistical models will be fitted with SAS statistical software (SAS version 9.4 of the SAS System, SAS Institute Inc., Cary, NC, USA).

Sample size calculation was performed with PASS statistical software (PASS Sample Size 2020 - NCSS, LLC).

### 2.6 Study procedures

#### 2.6.1 Consent and accrual

The study will be conducted once approval is granted by Local Ethical Committees, which will include consent to re-use administrative healthcare system data (in the informed consent patients will be asked whether in addition to the collection of clinical data from notes and other clinical sources they agree with the use of national registries (applicable for Italian Region of Lombardy), to collect further information in case they are lost to follow up) and access to data collected from mobile devices, sensors and other existing datasets for future research. Patients will be asked to sign an informed consent form that will allow them to accept or decline to participate and to use the developed monitoring tools. The informed consent will also include the possibility to share de-identified data according to *General Data Protection Regulation (*GDPR) and the results of their QoL questionnaire, which will be anonymized and made freely available on a public repository after study conclusion (e.g., Elixir network).

In this research study, the data that will be collected will be the minimum data set necessary to address the study endpoints, optimizing data quality, and participant privacy.

#### 2.6.2 Healthy volunteers

Before starting the study recruitment, at least 20 healthy volunteers (either researchers or collaborators) will have been enrolled for beta-testing of the app outside this clinical study.

#### 2.6.3 Quality of life data and follow up information

Quality of life information related to enrolled participants will be collected through validated questionnaires: EORTC QLQ-C30 and EORTC QLQ-HN43. Health status will be measured using the EQ-5D-5L, coping behavior using the CBI-B, and one PREM. Specifically, the PREM will be a study-specific question, asking patients “Are you satisfied about the care you have received during the follow-up?” (the possible answers will be “Absolutely disagree (I am not satisfied at all)”, “Moderately disagree”, “Neither agree nor disagree”, “Moderately agree”, “Absolutely agree (I am very satisfied)”). Questionnaires will be collected at study entry and after 6-, 12-, 18-, 24-month from randomization.

Patients’ follow-up information will be collected after treatment termination and will be updated at each evaluation. Data related to the disease outcome (overall survival, OS; disease-free survival, DFS) will be collected up to 24 months from randomization (**Table 4**).

**Table 4:** Study flowchart.

Procedure/evaluation	Randomization	Continuous (up to 24 months from study entry)	Months +6, +12, +18, +24 ( ± 2 weeks)
Informed consent	✓		
Baseline evaluation	✓		
Randomization	✓		
Demographics	✓		
Concomitant medications and medical events review	✓		✓
Clinical data retrieval (stage, pathology, HPV status)	✓		
Physical/emotional/social monitoring apps	✓	✓*	✓
QoL questionnaires	✓		✓

*for intervention arm only.

#### 2.6.4 Timing of QoL questionnaires completion, PROMs and PREM

Upon informed consent signature, at study entry, participants will complete baseline QoL questionnaires and will be randomized to receive the study BD4QoL platform or not. During their follow-up, which will be conducted according to international guidelines [e.g., ESMO (18, 19)], participants will complete further QoL questionnaires as scheduled in the study flowchart reported in **Table 4**: at randomization; +6 months ± 2 weeks from randomization; +12 months ± 2 weeks from randomization; +18 months ± 2 weeks from randomization; +24 months ± 2 weeks from randomization.

#### 2.6.5 QoL questionnaire data entry and data retrieval

The completion of study questionnaires will be performed the same day of the outpatient consultation ± 2 weeks, using web-based forms available at participating Centers or remotely, e.g., through tablets or personal computers (PC). QoL data will be accessible to clinical investigators, and to patients whenever asked.

If the subject does not go to the clinical institution for an outpatient visit or is not being seen every 6 months (± 2 weeks), the investigators will contact the patient by phone asking to fill in the requested questionnaires through the same web-based forms. The PoC dashboard will notify the investigators with an alert 2 weeks after the “due date” if any questionnaire has been initialized but has not been completed.

#### 2.6.6 Electronic alerts and subsequent interventions

As anticipated in the introduction, if the participants report or are identified as having a significant finding during monitoring, a specific set of interventions will be applied. This is particularly important since this study is not simply identifying potential problems which would lead to decrement in quality of life it is also about intervening in an earlier stage before the quality of life decrement takes place and thus maintaining quality of life. Among the data collected by the BD4QoL App (**Supplementary Materials**), physical activity (measured through step count), non-sleep activity (through sensor-based data), and social activities (measured as phone usage and movements as recorded by smartphone GPS) will be analyzed on a daily basis, and their variations will be used to activate alerts that will be sent to the study participant through the chatbot integrated into the mobile application, and to the PoC web application (**Supplementary Materials**). Based on the activated alerts, specific interventions will follow:

- Based on the behavior alteration: after the first behavior alteration is identified, the chatbot is activated and the alert is recorded in the PoC, without any notification to the clinical investigator. If the same alteration occurs again and the patient has a health issue, the alert is generated and notified in the PoC. If the same alteration occurs by the third time, the alert will be generated and notified in the PoC, independently of whether the patient has answered or not to the chatbot.
- Based on symptom identification: after the first symptom is reported, the chatbot is activated. If the symptom has low priority, the alert will be recorded at PoC without notifying the clinical investigator; if the same symptom occurs once more, the chatbot is activated again, and it will ask the patient whether they would like their healthcare provider to be notified; after the third iteration of the symptom, a notification to the PoC will be sent.

The iteration count is advanced only for consecutive events linked to the same domain and symptom.

#### 2.6.7 Study interruption and withdrawal

Participation in the study will be interrupted in case of any of the following conditions apply: death; disease recurrence; diagnosis of second primary malignancy; referred to another center; unable to perform follow-up visits (e.g., due to comorbidities); the participant asked to exit the trial, but allowed the research team to keep patients’ data that they already have collected to be used for research analysis; the participant withdrew his/her consent to the whole study, and wants his/her data to be deleted forever for any future analysis; the participant withdrew his/her consent to the whole study, and wants his/her data to not be used for any future analysis.

Moreover, the participant will discontinue the study when a decrease ≥10 points of global health-related EORTC QLQ-C30 QoL [clinically meaningful deterioration, as defined in (13, 14)] is detected between two questionnaires completed 6 months apart. This will be considered an event for the primary endpoint.

#### 2.6.8 Data management workflow

Within the BD4QoL platform, the data collection process is done at different levels:

- Clinical data. CRF data from REDCap.
  ○Data collected at study entry, after every 6 months (+/- 2 weeks) and also at unplanned visits that may occur during the trial.
  ○CRF data formats are also included in **Supplementary Materials**.
  ○Data is transferred at the moment of data entry from REDCap to the central BD4QoL repository.
  ○Data managers will be provided with credentials to access the REDCap tool for data entry. Different roles will be assigned depending on the person’s access (e.g., possibility to edit a patient, to lock a form, etc.). User accounts are managed directly through REDCap.
- PoC management: alerts generated, actions performed for alerts, patient enrollment and follow-up details, clinical visits report
  ○Data collected during the trial, when a new patient comes, and once an event occurs.
  ○PoC data-related formats are included in **Supplementary Materials**.
  ○No data transfer is needed as data is directly stored in the central BD4QoL repository.
  ○Healthcare professionals will be provided with credentials to access the PoC tool. User accounts are managed through the authentication server allocated in the BD4QoL platform. Different roles can also be assigned.
- QoL questionnaires. Study questionnaires are delivered through web-form.
  ○Data collected at study entry, after each 6 months (+/- 2 weeks) and also at unplanned visits that may occur during the trial.
  ○Answers of this questionnaire will be stored in the central BD4QoL repository as raw data, and the scoring of the questionnaires will also be stored in the same location.
  ○No data transfer is needed as data is directly managed in the central BD4QoL repository.
  ○User access for patients is based on the same credentials for the user accounts created at PoC. Only patients with authorized accounts are able to fill in the web form questionnaires. Physicians (with their PoC accounts) will also be able to fill answers on their patients’ behalf, to deal with exceptional circumstances such as the patient has filled in the questionnaire on paper for some reason (i.e., internet breakdown not letting the completion of the web-based forms).
  ○Healthcare professionals will assist patients if needed in the process of filling in the web forms.
- Patients’ physical, social and non-sleep domains through mobile App (further details are reported in **Supplementary Tables**).
  ○Data collected every day or every minute, depending on data category.
  ○Data formats for each domain are in **Supplementary Materials**.
  ○No data transfer is needed as data is directly stored in the central BD4QoL repository.
  ○The patients will install two applications, the foreground application for continuous passive data collection and the main BD4QoL mobile application, using the credentials from their user accounts, as set at PoC. Only authorized accounts stored in the central BD4QoL repository will be able to login and fill in the mobile app.

#### 2.6.9 Data monitoring and data quality assurance

Data quality procedures have been devised to ensure data verification and data validation. Data verification activities are used to monitor whether the mobile technology under study actually measures the data it claims to measure (i.e., steps, activities, light, phone usage information, and such). Data validation assures that the collected and subsequently processed data are “right”: they need to be suitable for the objective for which they are being collected.

During the CRF definition, data quality procedures for clinical data have been defined to ensure dependencies between variables and coherence during the data collection process through REDCap. In REDCap, all data dependency and consistency rules will be implemented. A complete data collection report with all the rules implemented and the issues found (if any) will be amended by clinical center data managers and updated in REDCap forms. The implementation of these validated rules will ensure the integrity of the data collection. Data will be transferred to the central BD4QoL repository once entered in the REDCap server. The central BD4QoL repository undergoes a periodical backup process to safeguard its content.

#### 2.6.10 Data security

The Data will be safely transferred and stored in the central BD4QoL repository hosted by Partner INETUM (Murcia, Spain). The central BD4QoL repository consists of a Data Hub managing the various databases needed to ensure the functioning of the BD4QoL platform. The Data Hub features processes that allow integrating, enriching, analyzing, and subsequently disseminating information based on the needs of different actors (details in **Supplementary Materials**):

- In the acquisition layer there are different tools that guarantee the incorporation of the data, regardless of their origin, volume, digital format, in a standardized format. This acquisition layer will provide mechanisms capable of making massive loads of datasets required by the project.
- A central set of databases, as well as their data management mechanisms inherent in each solution, make up the storage layer of the system. This set of databases is governed by the applications processes providing a set of algorithms, rule engines and analytics.
- The security of the system is provided as an additional layer, transverse to the system, which exposes the information and operations only and exclusively after it has validated an authorized access.
- The communications used for the exchange of messages between the system’s applications are done using the Hypertext Transfer Protocol Secure (HTTPS) protocol over the Transmission Control Protocol (TCP). The TCP protocol natively includes features such as error checking and acknowledgement interchange between peers, which means that if data is corrupted or not received, it is resent by the sender to ensure that it is received correctly. The BD4QoL Data Hub is deployed in the datacenter that INETUM has located in Murcia, Spain. This datacenter has tier IV certification, meaning that it includes capabilities to ensure data safety and availability. The characteristics of this datacenter at different levels are detailed in **Supplementary Materials**.
- For the cloud, specific backup and disaster-recovery policies are foreseen in case of corruption or detection of errors.
- For the mobile App, it is possible to identify if some data are missing at the stage of data collection. If this happens, it would be impossible to reconstruct the past in a digital form. If data are collected but the transmission to the cloud shows anomalies, such as data not transmitted when needed, but after a delay, specific scripts are foreseen to allocate the data in a way close to reality.

The above-mentioned technical measures ensure the integrity of the data collected in the BD4QOL platform, assuring that neither any corruption nor any data loss will occur. INETUM will not use patients’ data for purposes outside this research study.

Daily backups of the BD4QoL databases will ensure data reconstruction. The mobile apps have a data log (timestamp) for data collected and transmitted to the Data Hub. The main challenges faced here involve the following two challenges:

1. Data from the mobile device not fully collected due to failure of the process: the mobile app can perform a procedure to detect when data collection has stopped. This can happen in various situations, such as the device being offline, orwith insufficient memory space to locally store the collected data, or that the stream listeners for the sensors have unexpectedly stopped, or that connection to a 3rd party physical activity data collection app (e.g., Google fit) is stopped. Although “backend” procedures can detect after a certain period of time that data collection has been stopped, it is not possible to “reconstruct” past behaviors and thus all collected data from the past.
2. Data from the mobile app not adequately transmitted to the Data Hub: The mobile app involves a procedure to detect whether data from the mobile device are adequately transmitted to the Data Hub.
  - If no data is transmitted, this may be due to failure to connect to the Internet. When the mobile device is connected to the Internet, then the transmission will be restored.
  - In case of connection availability but data is not transmitted to the Internet, then these may be transmitted “all at once”, due to several issues specific to the mobile device itself. In this case, a “micro service” has been included to “allocate” the collected data to the estimated “right timestamps”.

### 2.7 Study management

#### 2.7.1 Patients’ data protection and privacy

All data within BD4QoL shall be handled using tools and processes with ‘privacy by design’ as the mindset. In general, the security principles of “need to know” and “least privilege” will be applied while determining access rights and privileges. The project will share data, with applied internal encryption, with the subject’s local identifier recorded in a hashed ID code. The list matching patient personal data and study ID will be stored offline in the clinical center which collects the data, which will manage it in compliance with relevant local legislation (e.g., some UK centers will store this document on a restricted access drive on their hospital NHS IT system). The personal data will remain at the recruiting center and will not be sent to the BD4QoL project team.

Participating hospitals are responsible for maintaining the anonymity of participants’ data collected from the medical notes and for safely storing and preventing unnecessary access to any information which may disclose the patient’s identity. The provisions of the above-mentioned GDPR will be adopted or – if more restrictive – national regulations in matters of personal data protection and privacy. Sensitive data (e.g., date of birth, date of diagnosis, date of follow-up visits, date of recurrence, date of death) will be used to track health-related data and will not be registered or used for the analysis

Data that leaves the participant’s phone will be: activity, non-sleep, telephone usage.

For those who provide a specific consent, the following data will be added to the list of data leaving the phone: visiting places related with sports, eating/drinking, nutrition, shopping, medication, traveling, finances, culture, religion, self-care, spirituality, education, sentiment, depression, or heath.

Details about how these data will be recorded by the BD4QoL platform are provided in **Supplementary Materials**.

A specific data protection impact assessment (DPIA) was provided by PI and the Data Protection Office (DPO) of the Study Sponsor (INT, Milan, Italy) on the 25^th^ June 2021, before the Ethical Committee approval.

#### 2.7.2 Access to data and database protection

Clinical investigators (the local PI and the delegated sub-investigators) will be allowed through an authorization list to access clinical and study-generated data from patients recruited at their own Center. Access to patient’s data, both anonymized and for clinical use (identifiable, as per current clinical practice), will only be granted according to each hospital regulations and restrictions (i.e., only authorized personnel that has been granted access by the patient or healthcare operators in charge of emergency interventions), and then local hospital regulations will be applied. Nonclinical study investigators will be able to access only pseudonymized data (both clinical and device-generated). Upon request, the investigators might access raw and processed unidentifiable data in the framework of the exploratory analyses mentioned above.

#### 2.7.3 Source data and patient’s files

For the eCRF completion, the source documents will be the medical records where the available demographic and medical information of a patient has to be documented. It should be possible to verify the inclusion and exclusion criteria for the study from the available data in this file. It must be possible to identify each patient by using this patient file. Additionally, any other documents with source data, especially original printouts of data that were generated by technical equipment, have to be filed. All these documents have to bear at least patient identification and the printing date to indicate to which patient and to which study procedure the document belongs. The medical evaluation of such records should be documented as necessary and signed/dated by the investigator.

#### 2.7.4 Investigator site file and archiving

The investigator will be provided with an Investigator Site File (ISF) at the start of the study.

This file contains all relevant documents necessary for the conduct of the study. This file must be safely archived after the termination of the study.

It is the responsibility of the investigator to ensure that the patient-identification sheets are stored for 10 years beyond the end of the clinical study (defined as last patient out). All original patient files must be stored for the longest possible time permitted by the regulations at the hospital, research institute, or practice in question. If archiving can no longer be maintained at the site, the investigator will notify the Sponsor/Representative of the Sponsor.

#### 2.7.5 Data management after study end

The investigator must retain all study records and source documents for the maximum period required by applicable regulations and guidelines or institution procedures, whichever is longer (minimum 5 years). The investigator must contact the coordinator Center prior to destroying any records associated with the study. If an Investigator of a participating Center withdraws from the study (e.g., relocation, retirement), the records shall be transferred to a mutually agreed upon designee (e.g., another investigator, IRB). Such transfer shall be reported in writing and notified to the Coordinating Center and to each Ethical Committee of the participating Centers.

Trial participants assure that the key design elements of this protocol will be posted in a publicly accessible database such as clinicaltrials.gov. In addition, upon study completion and finalization of the study report, the results of this study will be either submitted for publication and/or posted in a publicly accessible database of clinical study results. Patients will be informed of this option during the informed consent procedure.

#### 2.7.6 Quality assurance and safety

This study is to be conducted in accordance with the ICH Note for Guidance on Good Clinical Practice (ICH, Topic E6, 1995) dated July 17, 1996.

The representatives of the Clinical Quality Assurance Team of the sponsor are permitted to inspect the study documents (study protocol, case report forms, study medication, original medical records/files), as well as representatives of national regulatory authorities. All patient data shall be treated confidentially. In line with ICH GCP guidelines, monitoring will be the responsibility of the study sponsor, and it will include the verification of data entered in the eCRFs against original patient records. This verification will be performed by direct access to the original patient records and the monitoring staff guarantees that patient confidentiality will be respected at all times. The study protocol, each step of the data-recording procedure, and the handling of the data, as well as the study report, shall be subject to monitoring activities. Audits can be conducted to assure the validity of the study data.

Each participating site will maintain appropriate medical and research records for this trial, in compliance with Section 4.9 of the ICH E6 GCP, and regulatory and institutional requirements for the protection of confidentiality of subjects.

The Principal Investigator is responsible for ensuring that all staff involved in the study are familiar with the content of the protocol and trained regarding study procedures. Moreover, all the investigators involved in the trial are responsible for patient safety, and all events potentially related to patients’ safety must be reported in a timely, accurate, and complete manner.

## 3 Discussion

HNC survivors face many difficulties in implementing self-management in their daily life (34) (e.g. grappling with having to self-manage, interpreting self-management) and must fight personal, health-related and structural barriers (e.g. access to appropriate health services). They exhibit highly individualized approaches to self-management that often fail to meet their own specific needs. This has obvious impacts on their health, anxiety and QoL and even more on healthcare and social costs. GPs and welfare services are not fully included into HNC post-treatment management nor have direct and coordinated links with the specialists engaged in survivors’ follow-up at the cancer center. Physicians have limited insight on patients’ perceptions of QoL, based on few data collected during follow-up visits from patients’ interviews and – depending on hospital workflow – through structured Patient Reported Outcomes/Patient Reported Experience Measurement questionnaires (PROM/PREM) that measure body functions and health and psychological symptoms In this context, PoC specialists can only intervene when a late effect is reported or clinically diagnosed. So far, individual QoL trajectories have not been studied. Physicians are therefore applying standardized follow-ups which may delay recognition of late effects, and thus effective treatment.

The BD4QoL study is aimed at avoiding the deterioration of all HNC survivors, independently of the site of origin of their disease. Although the range of recommendations included in the chatbot is the same for all patients, we anticipate that the conversations will differ based on primary tumor site. However, the primary endpoint is the same for all HNC patients: preventing a clinically meaningful deterioration in subjectively-recorded global health status. This is still an unmet need for all HNC patients, and with this project we did not want to miss the opportunity to reach this ambitious goal avoiding a fraction of HNC survivors due to their primary tumor site. To reduce the heterogeneity of the patient cohort, we decided not to include patients with mucosal melanoma (also for their very high risk of recurrence), sarcoma, lymphoma, and thyroid cancers.

Understanding and addressing individual survivor’s needs, interpreting signs and symptoms of survivors’ health and psychological status is paramount in head and neck cancer where timely interventions can make the difference in individual patient’s QoL (35). However, despite instruments and tools for QoL monitoring, such as e-PROMs/e-PREMs (36), have demonstrated effectiveness for QoL improvement, difficulties and barriers hinder their practical use.

In the context of patient counseling, unsolved problems are the following:

- Healthcare professionals do not have the time that would be needed to counsel patients properly. Slots for outpatient visits are pre-specified and usually their duration is insufficient to efficiently capture the global survivors’ status. The standard oncological follow-up visit should mainly be focused on recognizing disease recurrence and/or major treatment-related late toxicities Indeed counselling is delivered within this scenario. All non-cancer and/or treatment related issues are referred to general practitioners.
- They do not have easily at hand the information that would be needed to counsel patients properly. The variety and the severity of reported signs and symptoms are so wide that they exceed the oncologists’ knowledge to effectively counsel patients, except for the classical and expected outcomes.
- They are not able to provide patients counselling with the necessary continuity. Between visits, patients are referred to general practitioners and specialists from other disciplines. Given this type of health care organization there is a substantial interest in creating and implementing cancer survivorship care as the one that can be offered in survivor’s clinics.
- Lack of information technology literacy may constitute a significant barrier for patient communication, self-management and coping strategies.

In this project, we aim to address survivors’ and physicians’ needs and to overcome the cultural, psychological organizational and technological barriers to systematic and coordinated monitoring of HNC survivors’ QoL.

Big data analysis might pave the way to new innovation-technology (IT) tools that leverage artificial intelligence (AI) techniques (e.g. machine learning) to infer more meaningful information from patients’ follow-up after treatment.

In this context, the writing of this protocol followed the EQUATOR (Enhancing the QUAlity and Transparency Of health Research) guidelines for clinical trial protocols for interventions involving artificial intelligence (the Standard Protocol Items: Recommendations for Interventional Trials – Artificial Intelligence, SPIRIT-AI Extension) (37) and according to the Clinical Trials Transformation Initiative (CTTI checklist) (38).

## 4 Ethics and dissemination

### 4.1 Protection of individuals/patients enrolled in the trial

The responsible Principal Investigator will ensure that this study is conducted in compliance with the protocol, following the instructions and procedures described in it, adhering to the principles of Good Clinical Practice and with current local legislation, and in accordance with: General Data Protection Regulation (GDPR), ICH (International Council for Harmonisation) Harmonized Tripartite Guidelines for Good Clinical Practice, Directive 2001/20/EEC of the European Parliament and of the Council, Declaration of Helsinki concerning medical research in humans (Helsinki 1964, amended Tokyo 1975, Venice 1983, Hong Kong 1989, Somerset West 1996 and Edinburgh).

### 4.2 Ethics and regulatory review

This trial will be initiated only after all required legal documentation has been reviewed and approved by the responsible independent ethical committee (EC) of the center according to all applying national and international regulations. Prior to patient participation in the trial, written informed consent must be obtained from each patient according to ICH GCP and to the regulatory and legal requirements of the participating country. Each signature must be personally dated by each signatory, and the informed consent and any additional patient information form retained by the investigator as part of the trial records. A signed copy of the informed consent and any additional patient information must be given to each patient or the patient’s legally accepted representative. This clinical study protocol was approved by the Sponsor’s Ethical Committee on 07/02/2022 (local study identifier INT267-21; PI dr. Carlo Resteghini) and at CSS on 09/03/2022 (local study identifier Prot N 20/CE; PI dr. Alfonso Manfuso)

### 4.3 Dissemination

The abstract of this protocol was submitted to ESMO (European Society for Medical Oncology) 2022 Conference. The final results of this clinical study will be published in impacted scientific journals and presented at international meetings.

## The BD4QoL consortium

Erlend I. F. Fossen^1^, Katherine Taylor^2^, Paul Nankivell^3^, Mriganke De^3^, Ahmad Abou-Foul^3^, Estefania Estevez-Priego^4^, Maria Fernanda Cabrera-Umpierrez^4^, Itziar Alonso^4^, Sergio Copelli^5^, Andy Ness^6^, Miranda Pring^6^ and Katrina Hurley^6^

^1^Oslo Center for Biostatistics and Epidemiology, University of Oslo, Oslo, Norway

^2^Division of Epidemiology and Health Care Research, JGU – Johannes Gutenberg University, Mainz, Germany

^3^Institute of Head and Neck Studies and Education, University of Birmingham, Birmingham, United Kingdom

^4^Oslo Center for Biostatistics and Epidemiology, Oslo University Hospital, Oslo, Norway

^6^Division of Oral and Maxillofacial Surgery - Bristol Dental Hospital, University of Bristol - Bristol Medical School, Bristol, United Kingdom

## Data availability statement

Further material about the study protocol are included in the article/**Supplementary Material**. Further inquiries can be directed to the corresponding author.

## Ethics statement

The studies involving human participants were reviewed and approved by Ethical Committee of the Fondazione IRCCS Istituto Nazionale dei Tumori, Milan, Italy and by the Ethical Committees of the clinical institutions enrolling patients. The patients/participants provided their written informed consent to participate in this study.

## Author contributions

SC, CV, ML, MM-S, AF performed the statistical analyses. SC wrote the first draft of the manuscript. All authors contributed to conception and design of the study, wrote sections of the manuscript, contributed to manuscript revision, read, and approved the submitted version.
