# Supplementary material for: A multicenter randomized trial for quality of life evaluation by non-invasive intelligent tools during post-curative treatment follow-up for head and neck cancer: Clinical study protocol
Source: Front Oncol. 2023 Jan 31;13:1048593. doi: 10.3389/fonc.2023.1048593 (PMC9927199; doi:10.3389/fonc.2023.1048593)
Supplement: Supplementary file 1 [file DataSheet_1.pdf]

# *Supplementary Material*

**Note:** the annexes (and their numbers) quoted in this supplementary text refer to the original study protocol approved by the Ethical Committees of the participating Institutions. The original protocol and the SPIRIT-AI checklist may be available to readers upon specific and reasonable request to the Authors.

## 1 BD4QoL platform

The BD4QoL ecosystem is composed of different applications and components that build up the required overall functionality:

- **BD4QoL Data management infrastructure**. This infrastructure allow the integration, enrichment, analysis and subsequently disseminate mation based on the needs of different actors of the following platform components.
- **The BD4QoL Data Hub** that acts as a master data repository for the project.
- **The mobile application** that collects data from the patient's smartphones and allows interaction with different metrics and results.
- **The Point of Care application**, a tool for health and social care professionals in the different Point of Care (PoC) engaged in patient's monitoring and follow-up.
- **Questionnaires' data collection**, a web form for collecting QoL, PREM and PROM data from patients.
- **The Patient Empowerment Platform** enables patients to hold two-way conversation interactions with the Bidi virtual assistant service providing non-clinical advice, the ability to send personalized notifications and collecting affective trait data.
- **IBM Cloud** hosts the Watson engine services enabling the chatbot dialogs and analysis
- **The Behaviour Recognition System** is a set of algorithms and processes that analyze the data available from the patient and infer the activities realized and their importance in assessing the quality of life.
- **The advanced QoL prediction application** generates predictive models that, by analyzing the different information available from patients and retrospective studies, enable the forecast

of what the QoL will be like in the future and, therefore, allow caretakers to adopt corrective measures that can help to improve it.

The following sections provide the details of each one of the components of the overall BD4QoL platform.

## 1.1 Data management infrastructure

The characteristics of the infrastructure where all BD4QoL platform components are allocated are detailed as follows:

- Facility:
    - Tier IV. 99.999% availability.
    - Anti-seismic construction with insulated electromagnetic.
    - Redundant infrastructure for mission environments review.
    - 1.2 MW of power maximum in datacentre of high density.
    - Double electric ring with 2 UPS and 2 groups generators with 1-week autonomy.
    - GREEN IT "Base Design", being 50% more efficient in the consumption of energy.
- Security:
    - Specialized security personnel 24x7.
    - Intelligent indoor and outdoor video surveillance system with intruder detection.
    - Access to critical rooms controlled by facial biometrics (TI, MPOE, SOC, NOC, etc.).
    - Very Early Smoke Detection Air (VESDA).
    - Water extinguishing system mist, avoiding the evacuation of the data centre.
- Operation:

    - Customized 24x7 support backed by technical team of experts with presence onsite IT and Industrial staff.
    - ITIL, SSAE16, ISO, ICREA standards level 5.
  - Communication:
    - 2N end-to-end redundancy.
    - Two independent links with diversified access and connection to two neutral points (Telvent and Interxion).
    - Own public address, balanced between the two links.
    - Safety equipment Service Provider logic, with protection against DDOS attacks.
    - 2 Multi-Carrier zones (MPOE) for service providers with exterior fingerprint access.

### 1.2 BD4QoL Data Hub

BD4QoL Data Hub consists of the set of repositories and data stores needed by the project applications. There will be relational databases that allow an efficient storage of the structured information represented by the forms to capture prospective information. It will also include NoSQL warehouses that will facilitate the intake of semi-structured data such as telemetry from the devices carried by the patient or the dialogues of the conversational assistant. Finally, the infrastructure will have file stores that enable the upload or download of batch information and data exchange among different environments.

### 1.3 Mobile application

The mobile application performs an unobtrusive collection of the needed personal data (e.g., accelerometer data, GPS data, etc.). Such app represents the unique touchpoint with the patients, and it also integrates the e-coach chatbot user interface (UI) as well as a specific UI for the patients to manage their personal data protection functions, specifically to decide which data can be collected or which not. The application collects data from different sensors in the device in a way that is transparent and unobtrusive to the patient, while other data is collected through direct user interaction with the chatbot.

The mobile application, available to the participants on their mobile devices, supports the following functions:

- Continuous data collection from the device's available sensors and operating system (that is accelerometer, GPS, ambient light, Screen status and Wifi connection.). The data collection is performed every 1 minute, however, dependent on the specific device that participants are using, this may vary to every 5 minutes.
- Aggregated pone-related data collection from the smartphone device (that is Calls and SMS log as well as phone applications data usage), once a day
- Aggregated external data from Google Fit (Steps and Activities), once a day
- Self-management "e-coach", consisting of a patient empowerment, natural language-based chat-bot, offering to participants:
    - Visualization of their own QoL data and related trends, as collected and inferred by the platform, according to rules established with clinicians
    - Counselling on symptoms self-management and providing relevant suggestions and recommendations for guided self-help
    - Establishing a tailored communication channel with the clinician
    - Detecting affective traits relevant to the participant's QoL assessment (e.g., joy, sadness, anger, disgust and fear together with a positive and negative sentiment towards certain keywords, concepts or entities) from the analysis of the dialog among the participant and the chat-bot, through natural language processing algorithms
- Direct management, on the part of the participant, of:
    - Consensus to the collection of the above-mentioned data, down to a "data element by data element" scale
    - Withdrawal from the study (in which case the PI is notified through the PoC support application)
- Dashboard reports on the data collected and analyzed at different timescale (per day, week, month or year) including graphical visualizations
- A micro service for automated data quality detection (in terms of data inconsistencies, missing data collected, transmission data faults, etc.)
- Temporary storage and subsequent network transmission of the data collected by the mobile app to the BD4QoL cloud infrastructure ("store and forward" approach), in order to be used in the PoC support application.

These features are delivered through four modules:

- **Administration module**: allows users to configure their personal details, such as data to be collected or permission options, as well as the setup of personal goals to be monitored.
- **Data collection modules**: these modules pick information from smartphone sensors and data from activities, i. e. calls and text messages logs.
- **Personal reporting modules**: these modules refer to dashboard reports that will be generated to demonstrate trends and analytical statistical data about one's health behaviour through the mobile app, including the achievement of trophies and medals for physical activities.
- **Bidi module**: integration with IBM Watson Assistant chatbot application.

### 1.4 Point of Care application (PoC)

The PoC application is a decision support system and workflow management system for clinicians dedicated to head and neck cancer follow-up, and will gather data from the CRF and the patients both through mobile app and patient inputted values (questionnaires).

The PoC application is delivered through the BD4QoL infrastructure, to be made available to clinicians at Point of Care and to investigators involved in the study. The application includes the following features:

- Link for collecting CRF data as described in Annex 10-14, through the RedCap application.
- Receiving pre-processed data collected by the mobile app and other data collected by the study that enter the risk stratification and prediction models described below, including data quality-related pre-processing (e.g., management of missing data, management of outliers, discarding low-quality data)
- Generating alerts, delivered to the participants through the mobile app's e-coach function, for low-risk events detected by rule-based data analysis algorithms established by clinicians, that warrant self-management actions.
- Generating alerts, delivered to clinicians through relevant notifications within the PoC support application, for moderate/high-risk events detected by rule-based data analysis algorithms established by the clinicians, that warrant medical attention.
- Visualization Dashboards to present the previously mentioned inferred features, as well as QoL questionnaire items and alerts, including temporal trends where relevant, as additional information resources for clinicians to assess participants' QoL trajectories.

More details can be found in Annex 19.

## 1.5 Questionnaires' data collection

Questionnaires' data collection app is a web-form tool containing all the QoL, PREM and PROMs to be filled-in by patients.

## 1.6 Patient Empowerment Platform

The Patient Empowerment Platform is composed of the chatbot engine that establishes a direct communication interface with the patient in order to know the status of the patients first-hand, by means of detecting relevant behavioural changes that might be indicative of their recovery through the sensors of their mobile devices. This tool allows collecting first-hand information on the affective and the health status of the patient, thus empowering and supporting in managing their symptoms aiming to improve their quality of life. While conversing with the patient, the e-coach analyses the conversations at times to find out more about the patient's affective state. It uses deep learning to extract metadata from the patients' messages producing artefacts such as entities, keywords, categories, sentiment, emotion, which are then stored for further analysis by the Point of Care or the the Bahaviour Recognition Systems. The e-coach service interacts with the BD4QoL Data Hub to store and retrieve relevant data captured in conversations with the patients together with the results of invoking the IBM Watson cognitive services to detect affective traits and risk models. Other data structures to manage may include text-based, time-dependent conversation sessions, reporting data from patient, Q&A conversations, actions resulting from said conversations, and pre-programmed interventions involving mobile phone push notifications. Eventually, it may also include a diary-like text feature with Natural Language Understanding analysis, or other similar approaches, of captured entries and a module looking at linguistic styles included in conversations between the chatbot and the patient.

The human-AI interaction is based on a patient counselling strategy outlining the clinical and technical terms of the e-coach interactions and the aim of the overall conversation-led platform. It provides the guidelines for the areas of the quality of life measured by the chatbot and feasibility for various types of measurements as per existing literature.

## 1.7 Behaviour Recognition System

The behaviour recognition system detects the activities performed by the patient and the behavioural markers that are important to measure their quality of life. It is an offline process that will do batch processing of the data retrieved from BD4QoL Data Hub.

Using pattern recognition datamining techniques and semantically annotated geospatial information, it will look for patients' behaviours. The results of this algorithm will be stored back in a relational store inside the Data Hub. The results will be used as the input for the Advanced QoL prediction models. These results may be used later by Point of Care application and the eCoach service for patient diagnose and empowerment.

## 1.8 Advanced QoL prediction models

The Advanced Quality of Life prediction tool calculates a patient's quality of life from the different data collected (mobile device sensors, call records, detected activities, behavioural markers, etc.). This value is compared with the values obtained at previous times and, if any of the defined conditions are met, it notifies the alert for risk of a drop in quality of life. This tool interacts with the Data Hub by retrieving the results of the benchmark models built-up from retrospective data, the prospective information from the PREM/PROM questionnaires, the inferred behaviour models and the data obtained/generated by the mobile application and using all these datasets to train its advanced prediction models.

## 1.9 Data management workflow

The workflow of the data generated and transferred within the BD4QoL platform is shown in Figure 1.

First, patient eligibility criteria are filled in REDCap. Then, the patient is randomized after checking patient compliance with the criteria. Once the patient is created in REDCap, it appears in the PoC and the healthcare professional enrols the patient by adding the patient mail. At PoC, the data manager can continue entering the study data entry for the patient. In the subsequent visits, all the clinical data will be entered through REDCAp. CRF data will be first stored in the REDCap server, allocated at UPM, and then transferred (in almost 'real-time') to the BD4QoL Data Hub. Patient enrolment data (i.e., start date, end date, enrolment status and mail) are stored directly in the BD4QoL Data Hub.

Second, the patient account is set up through the QoL web-form tool. At this moment, the patient, whose username is the study_id created in REDCap, will be able to change the password. At this

moment, the patient can fill in the questionnaires for the baseline period. For the follow-up PoC visits, the same tool will be used for inserting the questionnaires' data. Questionnaires' answers and the scoring automatically calculated by the tool are directly transferred to the BD4QoL Data Hub.

For the patients allocated in the intervention arm, the mobile app will be installed and set up. Details about this process are included in Annex 16. Data monitoring starts at this time, and this data collection (also detailed in the annex 16) is directly stored in the BD4QoL Data Hub.

Data from questionnaires, CRF and data collected from the mobile app will be available for physicians consultation through the PoC tool. Also, this data will feed the behaviour recognition system and the advanced QoL prediction models, both handling the data in the BD4QoL platform. In addition, behaviour data reporting will also be available to each individual patient through the mobile app.

The Chatbot will have access to the patient conversation text and necessary data (without Personal Information) to perform affective traits and symptom analysis through the chatbot functionality integrated in the mobile app. No other applications text will be analysed. All chatbot messages will be processed by  services running on the BDQoL Data Hub and Watson services on IBM Cloud in-flight  and transferred to the BD4QoL Data Hub for permanent storage.

# 2 Project data

## 2.1 Data design

Data are divided in 5 main areas:

- Core: The core classes and instances .
- Behavior: The classes and instances used to describe the behavior of the patients.
- Affective: The classes and instances used to describe the emotional and affective status of the patients.
- Quality of life: The classes and instances used to describe the quality of life of the patients.
- Head and neck cancer: The classes and instances used to describe the head and neck cancers.

The relations between the different classes can be seen in Figure 1. The designed classes and instances for each of the main areas can be found in the appendices I to V.

The class tables have the following columns:

- Class: The class that is being described.

- Property: Properties that compose that class.
- Type: The type of the properties, they can be either primitive values or other classes.
- Explanation: An explanation of the class and what it represents.

Not all the classes have predefined instances in the data design, some of the classes are intended for instances that will be created during the execution of the project:

- Instance: The name of the instance.
- Class: The class that it is an instance of.
- Property: The name of the property.
- Value: The value of the property.

### 2.2 Core

It is composed by the following classes:

1. Person: The class representing the patient.
2. Data_source: The data sources used to validate different classes and instances.
3. Demographic_clinical_data: Summarizes the clinical and demographic data of a person/patient.
4. Risk_factors: Summarizes the risk factors information of HNC of a person/patient.

### 2.3 Behavior

It is composed by the following classes:

1. Activity: Activities related to the QoL of H&N cancer survivors
2. Activity_domain Domain to which specific activities belong to.
3. Low_elementary_action: Low Elementary Actions related to the QoL of HNC survivors.
4. Expert_activity_model: Expert Activity Models used to detect the execution of activities based on the executed actions.
5. Executed_activity: An activity detected using the Hybrid Activity Recognition System.
6. Executed_action: Low Elementary Actions executed by the patients.

### 2.4 Affective

It is composed by the following classes:

1. Specific_emotional_status: List of the possible emotions that can be detected (Emotional behaviour /emotion process - detectable by Watson NLU)
2. Specific_composite_emotional_status: List of possible composite emotions. (composite emotional behaviour/emotion process - detectable by Watson NLU)
3. Subjective_emotional_feeling: List of possible subjective emotions directly dependent and variant for each patient.
4. Physiological_ response: List of physiological responses that could be detectable using the smartphone or wearables.

### 2.5 Quality of Life

The QoL classes model the patients perceived quality of life. These are based on stablished QoL and PREM/PROM questionnaires. It is composed by the following classes:

1. PRO_score_QoL: Score for the scales and items as measured by the specified questionnaire.
2. PRO_ question_QoL: Specific QoL questionnaire questions.
3. PRO_health_status: Perception about the patient health status.
4. PRO_Metrics_User: Metrics for each patient.
5. Pro_Behaviour: patient's opinion on their ability to carry out specified behaviors.

## 2.6 Head and neck cancer

It is composed by the following classes:

1. Clinical_T_and_N_characteristics: The class summarizes the clinical T and N characteristics of the tumor of a person/patient.
2. Pathology_data: The class summarizes the pathology data of a person/patient.
3. Chemotherapy: The class summarizes the information of chemotherapy treatment of a person/patient.
4. Radiotherapy: The class summarizes the information of radiotherapy treatment of a person/patient.
5. Follow_up: The class summarizes the follow-up information of a person/patient.
6. Toxicity: The class summarizes the toxicity information of a person/patient as result of treatment.
7. Symptoms_last_evaluation: The class summarizes the information related to person/patient symptoms at last evaluation.

# 3 Activities and data types detected through devices

## 3.1 Mobile Applications and data collected

To allow personal behaviour data collection, the study participants will setup two mobile applications at a "setup study visit" in the PoC. These applications will be the following:

a. a foreground application that will collect data every 1 minute from the user's smartphone sensors, as follows:

   1) **Light** of the surrounding where the mobile smartphone device is located (calculated in number of LUX units)

   2) **Accelerometer** of the participant's position (measurement in "X", "Y" and "Z" number)

   3) **Screen** status of the smartphone (possible values include SCREEN_ON, SCREEN_OFF, UNLOCKED)

Once installed, the user will no longer need to have any interaction with the application itself, neither to re-launch or re-open it. Data is continuously connected (even at plane mode) and is transmitted to the data hub when internet connection is restored. The application initiates a notification to the user on the top left "notifications bar".

b. a mobile application with the following fundamental modules:

1) a data collection module based on "alarm-manager" technology that will collect data every 1 minute from the participant's smartphone device. This data is:

   i. **GPS** location (Latitude, Longitude) **[B1.1]**

   ii. **Wifi** connection (name of Wifi) **[B1.2]**

2) a phone data collection module that will collect once per day information about the following on a daily basis **[B2]**:

   i. **Calls** log: detailed collection of a report about calls made from – to the participant's phone. The information collected will describe whether the call is incoming, or outgoing or missed. In addition, the contact number involved will be collected in an encrypted form.

   ii. **SMS** log: detailed collection of a report about sms' made from – received to the participant's phone. The information collected will describe whether the SMS is incoming or outgoing. In addition, the contact number involved will be collected in an encrypted form.

   iii. **Applications** Usage: data about all smartphone applications used will be collected. This information will include the name of the application as well as the time spent during the course of a day **[B2.3]**.

3) an external data collection module that will collect physical data from Activity Transition API and Google Fit API from the participant's smartphone once a day. This data will provide information about:

   i. Steps made from the smartphone user on a daily and hourly basis **[B3.1]**

   ii. Activity sessions, such STILL, WALKING, CYCLING, etc. made by the participant on a daily basis **[B3.2]**.

### 3.2 Operative system choice

As commented in the sections above, the main data expected to be analyzed within the context of BD4QoL are phone usage and sensor-based data, activities and sleep. Those domains cover specific data:

- Phone usage: phone calls, messages, phone applications, Wifi's connections.
- GPS, accelerometer, steps, screen, light.
- Activities: list of google activities.

- Sleep activity, not possible without wearable, so it is measured the non-sleep events rate.

Due to the iOS operative system, with iPhones, it is not possible to gather phone usage and some sensor-based data (accelerometer, screen, light). Also, the GPS frequency is not the same for iOS or Android. Due to these constraints, in the BD4QoL prospective study, only Android patients are recruited.

## 3.3 Volume of data collected

Data collected from the mobile application, as shown in Table above, is performed at different frequency according to the type of the data itself. Specifically, data is collected:

- every 1 minute for: GPS, Light, Accelerometer, Screen status, and Activity status
- once a day for: phone data (Calls, SMS, applications) and STEPS counter.

The above data can be collected offline (if no Internet connection is present) and will be sent to the data HUB when connection is restored. Normally, a full day of data would result to a maximum collection of 1440 minutes of data (24 hours, 60 minutes per hour). If however the clinical trial participant keeps the smartphone device OFF for a period of more than 30% of total daily time, i.e. for more than 7,2 hours (continuous or cumulatively), then this day will not be considered a "valid" data of data to be analyzed for the intervention alerting mechanism.

## 3.4 Setup Process

The setup of the two mobile applications will be performed within the PoC first visit using the participant's smartphone under the assistance and guidance from a trained person from the hospital.

*Prerequisites:*

1. The participant uses an Android mobile of OS version 7 or above.
2. The participant already has a google account (since an android device phone is used)
3. The participant has already installed Google Fit application from Play Store (Free) on the smartphone (if not, it should be installed and opened).

*Set-up steps:*

S1: Install (from a link) the foreground application (application "A" above)

S2: Settings for foreground application: Battery Optimization must be OFF for this application. Other special configuration guidelines may be applicable for specific brands and devices.

S3: Open the application (no other activity or interaction needed)

S4: Install (from a link) the alarm-manager application (application "B" above), named also as BD4QoL application

S5: Accept ALL Permission Notifications shown after installation is completed

S6: Go to Location Permissions in the BD4QoL application and navigate into “Location”. Make it “Allow all the time” – not only when using the application

S7: Go to Settings – Data Usage Access (or special access) and specifically make it ON for this application

S8: Go to Setting – Non Battery Optimized Applications and add the BD4QOL application

S9: Open the BD4QOL application

S10: Login with Study ID and password given

S11: Only for the first time, follow the instructions for “Sign in with Google” and complete Google Login

S12: Overview Settings and Permissions within the BD4QOL application

### 3.5 Daily Usage Process

On a daily usage, the participant will need to do only the following:

1. If the phone restarts, then the participant will need to open the application “B” again
2. The participant will need to connect at least once a day to Internet.

### 3.6 GDPR Compliance

The overall BD4QOL platform is developed in compliance with GDPR both “by design” and “by default”. Protection and compliance with policies from GDPR is ensured throughout the whole life cycle of the personal data, i.e. during the following stages:

a) Installation of the apps on study participants’ mobile phone devices

    - The PoC professional physicians will assist in the process of setting all necessary applications within the participants smartphone devices, under their supervision. The patients will always be allowed to see what the PoC professional performs with their phone.
    - The PoC professional will never ask for the patient his/her username and password for any account stored and operated in the phone other than the BD4QOL application, neither the UNLOCK pattern that may need to be drawn.
    - The PoC professional will not cable the mobile phone into any laptop – desktop or even server to download the necessary applications. All applications will be downloaded from a shared place (URL).
    - Before installing the necessary applications, the PoC professional will explain to the patient briefly the steps that will be followed and that no personal account details will need to be revealed what so ever.

- The PoC professional installing the applications to the patient's phone will be authorized to do so and a log of the installation process during the patient's visit will be made.
- The phone applications authorize the user based on a study ID number which is the account username. No information about the name, or the location of the patient.

b) Data collection and transmission to BD4QOL data hub

- Before data collection, the patient has been fully informed and given his/her consent about what kind of data are being collected, for how long, what are the main security measures taken to support their privacy, and when these can be deleted. Only patients that consent to participate in the study trial can use the mobile applications for the prospective study.
- Data from the mobile applications, both the foreground and the main application, are collected and annotated according to the user's study ID. This ensures the users' anonymity at both the mobile smartphone device and the BD4QOL data hub (server).
- The data from the mobile device are transmitted to the BD4QOl data hub in different intervals, some every 1 minute, others every three hours or once a day, according to the type of the data collected. Data transmitted reference to a specific study ID, not any personal information. Once transmitted, any locally cached data are being deleted for security reasons as well as resources optimization use.
- At any point, patients may withdraw from the data collection and prospective study trial with a clear and easy interface.
- Only the relevant personal data for the clinical study are being collected from study participants.
- The content of calls, sms, or other mobile applications is not collected. Only a log file when the person makes or receives or receives a call, or types or gets an SMS, or interacts with a smartphone application.

c) Data management in the BD4QOL data hub

- The data are treated confidentially, so that no one can identify a patient except by the identifier assigned to him/her, because no personal information is stored in the data hub.
- The data is stored with guaranteed integrity, so that the data can only be modified by those authorized to do so. Data need to be available for any consultation since it is stored, guaranteeing data consistency.
- The data of a patient who decides to leave the study are deleted at the time the patient makes the decision to leave the study. This will be made based on the withdraw options included in the adequate form through REDCap (more details in annex 10).

- The data hub makes it possible to obtain a copy of the data stored on a patient and to send it in a file when the patient requests it.
- As mentioned above, the information stored guarantees the anonymization of the data, so that the unequivocal identification of a patient is not allowed.
- The project database is secured, so that only authorized users can access it and perform certain actions depending on the access level associated with that user. Updates on patient's data can be performed from the patient him/her-self within the mobile app, in regard with Google account. No other personal data are stored in the device.

d) Data transfer to PoC visualization and re-identification of study participants to allow physicians' follow -up

- Data transfer from the database to PoC is done using the HTTPS protocol to ensure that data is not modified between source and destination and that data is sent encrypted.
- As for patient re-identification, this is a process that can only be carried out in the different hospitals participating in the study, because this is the place where the translation files for identification are located.

# 4 Point of care tool (PoC)

The PoC application is a decision support system and workflow management system for clinicians dedicated to head and neck cancer follow-up, that gathers data from the Head and Neck Cancer Survivors through the REDCap tool, the mobile app and the patient inputted values (questionnaires).

Specifically, it is a web-based tool designed for the management of research studies and operations, that allows clinicians to carry out daily activities and procedures focused on the follow-up of head and neck cancer patients. This tool provides clinicians with a series of dashboards for data visualization, to analyse and interact in an intuitive way with all the information gathered.

The PoC tool includes several functionalities to manage the BD4QoL data. Main functionalities are:

- Comprehensive visualization of the general information gathered for every single patient.
- Comprehensive visualization and navigation through the full trial study data collection.
- Interactions with an individual patient through notifications and REDCap synchronization.
- Classification and monitor of information from past visits, pending visits and planned visits.
- Management of patient alerts related to symptoms and behaviour.

All the user interfaces, interaction workflows and use cases are detailed in the following sections.

## 4.1 BD4QoL poc workflow design

The PoC tool application comprises five modules: The main patient information list, the trial dashboard, the exploratory dashboard, the visits management and the alerts management. First, the user logs in the application where the five modules are accessible:

- **Patient infromation module**: This module includes general data visualization for every single patient. The PoC user is able to check the clinical pathway of a patient from the list and may contact them either by phone or by scheduling a visit.
- **Trial dashboard:** This module includes general data visualization for the whole study trial, focused on statistics for demographic and specific health condition data. This module includes all the different centers.
- **Exploratory dashboard**: This module includes general data, focused on input data gathered from the mobile phone application, such as questionnaires, phone usage and affective traits.
- **Visits management**: This module lists all visits so the forms of each past visit are available, and the date of pending visits can be modified if necessary. Withdrawal report is also included in this module.
- **Alerts management**: In this module the user may check the history of alerts of all the patients and proceed with the most suitable interventions.

## 4.2 User Interface design

### 4.2.1 Visualizing the user login and access permissions

This functionality allows to access the PoC tool. The users of the PoC tool are registered by the administrator.

### 4.2.2 Patient Information

Once the users log into the PoC tool, they are provided with an overview of the Patient Information module. The main patient list is ordered by patients' id number and shows their enrolment date, latest updates, the study arm they belong to, their current status on the study (either *Active* or *Withdrawn*) and the eCRF data. The PoC users can enrol a new patient through the 'Data Collection (REDCap)' button and access every patient's information by clicking on a particular id.

There is a sidebar navigation menu in every screen of the PoC tool that provides access to the five main modules and a utility navigation menu on the top-right corner with essential functionalities as full-screen switch, notifications such as symptom alerts, and logout.

After clicking on a patient id, the screen with basic demographic data and the study trial conditions appears. There also is a drop down list with the results of the five questionnaires that patients have to fill when enrolling the study.

Within the same screen, scrolling down, the user can visualize the phone-based data collected in terms of steps, non-sleep events rates, phone usage and affective traits. This data can be visualized by week, month or year. There is also a 'help' icon for every chart with a short description.

Navigating through the menu of the Patient Information module, the user can also access the questionnaire report, visit report, alert report and communication history. At the Questionnaire Report, the user can check the data from previous questionnaires and inspect if there are any pending or outdated missing questionnaires.

At the Visit Report, the user can check the data from previous visits (orange icon) or fill in the form for a pending visit by accessing the REDCap platform through the edit option (grey icon). To schedule a new unplanned visit the user may click on the 'New Unplanned Visit' button and specify a date. Pending visit dates can be modified at any time.

At the Alerts Report screen, the user can check alerts generated from the BD4QoL mobile application. The list shows the date when the alert was generated, the frequency of appearance which influences the priority assigned, and the type of the alert, which can be related to social activity, physical activity or non-sleeping hours. The symptom level may be low priority (green), mid priority (yellow), high priority (red) or no symptom related (blue).

The last column indicates how the alert was managed, otherwise the message link 'Go to manage alert' appears. Here the PoC users can manage any pending alert for this specific patient (by email, call, sending a writing message or creating an unscheduled visit) and add pertinent comments in the 'intervention modal' screen that appears after clicking the link.

After selecting a management option, the status of the alert will automatically change to 'Complete' and the last column 'Action' will specify the intervention selected.

Finally, at the Communication History, there is a recap of visits and alerts. They are categorized by the Communication Channel which can be on a clinical visit or through generated alert, the date it was notified, the type of the alert and the actions performed as previously described.

#### 4.2.3 Trial Dashboard

The information presented on the Trial Dashboard includes information related to the clinical record pathway and the enrolment curve. All the data available for the study trial regarding scheduled visits and alerts is summarized here, and can be filtered by center. When scrolling down, the PoC user will find demographic data such as mean age and gender of the participants, tumour site and stage statistics, status of risk factors (smoking, alcohol consumption and comorbidities).

#### 4.2.4 Exploratory Dashboard

The Exploratory Dashboard provides an overview of all the data collected regarding questionnaires, physical activity, phone usage and affective traits. This data is the same data provided in the patient information but presented in an aggregated way and can also be filtered by last week, last month or year.

#### 4.2.5 Visit Management

The Visits Management dashboard provides a visualization of the clinical appointments scheduled for each patient, this includes both planned and unplanned visits. The appointments list is provided in a table that contains the main information of each visit such as the reason (i.e., planned or unplanned), the date, and the timing since the enrolment. The table can be filtered by any of these fields to facilitate the visualization of all the visits. Within the "All visits" view, an additional column presents the status of de visit indicating if it is complete or pending.

An option to edit the information of the visit is provided in both visualizations for the pending visits. When the "edit" icon is selected for any pending visit, a pop up message will appear to access the REDCap platform and edit or add the information of the CRF for the follow-up consultation (in the

case of planned visits) or the Contact In Between Or After Study Scheduled Visits form (in the case of unplanned visits).

In the case of the Withdrawal management, an alert appears in the bell icon of the top-right panel whenever a patient requests to withdraw and needs to talk to a physician, or if a patient has been logged out for at least two weeks. The message is 'The patient wishes to withdraw from the study' and can be accessed either by clicking on the alert or going to the Withdrawal Report at the Visits Management module.

Afterwards, the physician should contact the patient and schedule a visit. This visit is required to understand the motives of the patient that wants to withdraw and to fill in the appropriate REDCap formwhere it is specified how to proceed with the data registered so far. When the Withdrawal report appears as pending the PoC user can click on the 'edit' button to access this REDCap form, then the status will change to 'Confirmed' in red. Alternatively, if the patients log into the mobile application, the status will automatically change to 'Relogged' in green, and the REDCap form is no needed.

### 4.2.6 Alerts management

The alerts management provides a summary of all the alerts generated for every patient ID. This functionality can be accessed either through by the navigation drawer or by clicking on the notification bell icon located in the header of all the screens of the PoC tool. When a new alert is generated the bell icon changes, showing the number of alerts generated since the last access.

The visualization of the Alerts Management follows the same rationale as the Visits Management, being the alerts categorized as *Pending*, when new alerts generated that have not been managed yet, or *Solved*, when a physician has already handle this alert. By clicking either on the patient ID or the message 'Go to patient alerts' the PoC user will access the Alerts Report tab of the Individual Patient Data.

The Alert Management also includes the Messages Unrecognized by the chatbot. This table shows the patient ID who generated the message on a chatbot conversation, the date the message was registered, the type of message and the text itself. On the last column the clinician can mark as read those messages already reviewed.

## 4.3 Data managed in the PoC tool

Once the patient is enrolled in the study and the patient Study_ID is created in the REDCap platform, the PoC user fills in the eligibility form and the patient is allocated to the intervention or control arm. Then, the collected data are stored in the BD4QoL Data Hub.

## 5 Supplementary Tables

| Domain | Alert | Intervention |
|---|---|---|
| Physical<br><br>activity | 1. steps <50% from usual* in 2 weeks<br><br>2. steps <50% from usual* for further 2 weeks<br><br>3. steps <50% from usual* for further 2 weeks (total 6 weeks) | 1. the chatbot (Annex 8) is automatically activated and it asks the patient whether any health issue has occurred<br><br>- If not, then the chatbot automatically provides the patient with motivational tips, including evidence-based recommendations about physical activity (e.g., https://health.gov/sites/default/files/2019-09/Physical_Activity_Guidelines_2nd_edition.pdf). *Chatbot-related alert will appear in the PoC without any intervention required, only with an informative purpose. [blue-chatbot managed]*<br>- If yes, then the chatbot questions the patient about which health issue occurred and automatically provides the patient with related tips for managing the health issue. In addition:<br>● If the health issue is of low priority level the chatbot automatically provides the patient with motivational tips, including evidence-based recommendations about physical activity (e.g. https://health.gov/sites/default/files/2019-09/Physical_Activity_Guidelines_2nd_edition.pdf). *Low priority alert will appear in the PoC without any intervention required, only with an informative purpose. [low-chatbot managed]*<br>● If the health issue is of moderate priority level, the chatbot briefly questions the patient about additional information useful for the point of care and automatically contacts the point of care. *Moderate priority alert will appear in the PoC, without any intervention required, only with an informative purpose. [moderate-chatbot managed]*<br>● If the health issue is of high priority level, the tips given for managing it include a prominent recommendation to get in contact with the GP or the A&E department as soon as possible. *High priority alert will appear in the PoC and intervention will be requested. [high-PoC action]*<br><br>2. the chatbot automatically asks the patient again whether any health issue has occurred<br><br>- If not, then the chatbot automatically provides the patient with motivational tips, including evidence-based recommendations about physical activity (e.g., https://health.gov/sites/default/files/2019-09/Physical_Activity_Guidelines_2nd_edition.pdf). *Chatbot-related alert will appear in the PoC without any intervention* |

| | | |
|---|---|---|
| | | *required, only with an informative purpose. [blue-chatbot managed]*<br>- If yes, then the chatbot questions the patient about which health issue occurred and automatically provides the patient with related tips for managing the health issue. In addition:<br>● If the health issue is of low priority the chatbot asks the patient whether she would like to have a medical consultation.<br>- If the answer is yes, the chatbot briefly questions the patient about additional information useful for the point of care and automatically contacts the point of care. Nurses/Physicians will intervene as clinically indicated. *Low priority alert will appear in the PoC, and intervention will be requested. [low-PoC action]*<br>- If the answer is no, no further action is undertaken by the chatbot. *Low priority alert will appear in the PoC without any intervention required, only with an informative purpose. [low-chatbot managed]*<br>● If the health issue is of moderate priority level, the chatbot briefly questions the patient about additional information useful for the point of care and automatically contacts the point of care. Nurses/Physicians will intervene as clinically indicated. *Moderate priority alert will appear in the PoC, and intervention will be requested. [moderate-PoC action]*<br>● If the health issue is of high priority level, the tips given for managing it include a prominent recommendation to get in contact with the GP or the A&E department as soon as possible. *High priority alert will appear in the PoC, and intervention will be requested. [high-PoC action]*<br>3. the chatbot automatically asks the patient again whether any health issue has occurred<br>- If not, then the chatbot automatically provides the patient with motivational tips, including evidence-based recommendations about physical activity (e.g., https://health.gov/sites/default/files/2019-09/Physical_Activity_Guidelines_2nd_edition.pdf). *This will generate an alert in the PoC due to the reiterative nature of the alert (alert will not have assigned any priority level as no symptom is related to it). [blue-PoC action]*<br>- If yes, then the chatbot questions the patient about which |

| | | |
|---|---|---|
| | | health issue occurred and automatically provides the patient with related tips for managing the health issue. In addition:<br>● The chatbot also briefly questions the patient about additional information useful for the point of care and automatically contacts the point of care. Nurses/Physicians will intervene as clinically indicated<br>● If the health issue is of high priority level, the tips given for managing it include a prominent recommendation to get in contact with the GP or the A&E department as soon as possible.<br>● *Low, moderate or high priority alert will appear in the PoC, and intervention will be requested. [low/moderate/high-PoC action]* |
| Non-sleep activity | 1. NSER > +50% from usual* in 2 weeks<br><br>2. NSER > +50% from usual* for further 2 weeks<br><br>3. NSER > +50% from usual* for further 2 weeks (total 6 weeks)<br><br>* **Definition of "non sleep events rate" (NSER)**: proportion of total time (in minutes) for which SCREEN Status=ON has value, divided by the duration of the user selected nightly monitoring period | 1. the chatbot (Annex 8) is automatically activated and it asks the patient whether any health issue has occurred<br>- If not, then the chatbot automatically provides the patient evidence-based recommendations about sleep hygiene (e.g. https://www.cdc.gov/sleep/about_sleep/sleep_hygiene.html). *Chatbot-related alert will appear in the PoC without any intervention required, only with an informative purpose. [blue-chatbot managed]*<br>- If yes, then the chatbot questions the patient about which health issue occurred and automatically provides the patient with related tips for managing the health issue. In addition:<br>● If the health issue is of low priority level the chatbot automatically provides the patient with motivational tips, including evidence-based recommendations about sleep hygiene (e.g. https://www.cdc.gov/sleep/about_sleep/sleep_hygiene.html). *Chatbot-related alert will appear in the PoC without any intervention required, only with an informative purpose. [low-chatbot managed]*<br>● If the health issue is of moderate priority level, the chatbot briefly questions the patient about additional information useful for the point of care and automatically contacts the point of care. *Moderate priority alert will appear in the PoC, without any intervention required, only with an informative purpose. [moderate-chatbot managed]*<br>● If the health issue is of high priority level, the tips given for managing the health issue include a prominent recommendation to get in contact with the GP or the A&E department as soon as possible. *High priority alert will appear in the PoC and intervention will be requested. [high-PoC action]* |

| | | |
|---|---|---|
| | | 2. the chatbot automatically asks the patient again whether any health issue has occurred<br><br>- If not, then the chatbot automatically provides the patient with evidence-based recommendations about about sleep hygiene (e.g. https://www.cdc.gov/sleep/about_sleep/sleep_hygiene.html). *Chatbot-related alert will appear in the PoC without any intervention required, only with an informative purpose. [blue-chatbot managed]*<br>- If yes, then the chatbot questions the patient about which health issue occurred and automatically provides the patient with related tips for managing the health issue. In addition:<br>● If the health issue is of low priority the chatbot asks the patient whether she would like to have a medical consultation.<br>o If the answer is yes, the chatbot briefly questions the patient about additional information useful for the point of care and automatically contacts the point of care. Nurses/Physicians will intervene as clinically indicated. *Low priority alert will appear in the PoC, and intervention will be requested. [low-PoCaction]*<br>o If the answer is no, no further action is undertaken by the chatbot. *Low priority alert will appear in the PoC without any intervention required, only with an informative purpose. [low-chatbot managed]*<br>● If the health issue of moderate priority level, the chatbot briefly questions the patient about additional information useful for the point of care and automatically contacts the point of care. Nurses/Physicians will intervene as clinically indicated. *Moderate priority alert will appear in the PoC, and intervention will be requested. [moderate-PoCaction]*<br>● If the health issue is of high priority level, the tips given for managing the health issue include a prominent recommendation to get in contact with the GP or the A&E department as soon as possible. *High priority alert will appear in the PoC, and intervention will be requested. [high-PoCaction]*<br><br>3. the chatbot automatically asks the patient again whether any health issue has occurred.<br><br>- If not, then the chatbot automatically provides the patient with evidence-based recommendations about about sleep |

| | | |
|---|---|---|
| | | hygiene (e.g. https://www.cdc.gov/sleep/about_sleep/sleep_hygiene.html). *This will generate an alert in the PoC due to the reiterative nature of the alert (alert will not have assigned any priority level as no symptom is related to it). [blue-PoCaction]*<br>- If yes, then the chatbot questions the patient about which health issue occurred and automatically provides the patient with related tips for managing the health issue. In addition:<br>● The chatbot also briefly questions the patient about additional information useful for the point of care and automatically contacts the point of care. Nurses/Physicians will intervene as clinically indicated<br>● If the health issue is of high priority level, the tips given for managing it include a prominent recommendation to get in contact with the GP or the A&E department as soon as possible.<br>● *Low, moderate or high priority alert will appear in the PoC, and intervention will be requested. [low/moderate/high-PoC action]* |
| Social<br><br>activities | Either one or the other of the following conditions are the case:<br><br>1a. Phone usage > +/-50% from usual* in 2 weeks<br><br>1b. Average daily travel radius[1] < -50% from usual* in 2 weeks | 1. at the first occurrence of either 1.a and/or 1.b, the chatbot (Annex 8) is automatically activated and it asks the patient whether any health issue has occurred<br>- If not, then the chatbot recommends the patient to actively communicate with the chatbot in case any new symptom or health-related issue is detected. *Chatbot-related alert will appear in the PoC dashboard as a no-priority alert without any intervention required, only with an informative purpose for the PoC. [blue-chatbot managed]*<br>- If yes, then the chatbot questions the patient about which health issue occurred and automatically provides the patient with related tips for managing the specific health issue. In addition:<br>● If the health issue is of low priority level the chatbot automatically provides the patient with tips or recommendations on the specific health issue detected by the chatbot. *Low priority alert will appear in the PoC without any intervention required, only with an informative purpose. [low-chatbot managed]*<br>● If the health issue is of moderate priority level, the chatbot briefly questions the patient about additional information useful for the point of care and automatically contacts the point of care. *Moderate* |

[1] farthest radius from home traveled by any means in a day

| | | |
|---|---|---|
| | | *priority alert will appear in the PoC, without any intervention required, only with an informative purpose. [moderate-chatbot managed]*<br>● If the health issue is of high priority level, the tips given for managing the health issue include a prominent recommendation to get in contact with the GP or the A&E department as soon as possible. *High priority alert will appear in the PoC, and intervention will be requested. [high-PoC action]*<br>2. From the second occurrence of 1.a and/or 1.b (lasting at least 4 weeks) the cutoff for physical activity and sleeping alerting are changed to 30% instead of 50% to generate alert to patient (first 2 and 4 weeks) and then to point of care (after 6 weeks). *Chatbot-related alert will appear in the PoC without any intervention required, only with an informative purpose [blue-chatbot managed]* |

Supplementary Table 1. Alerts and interventions

** "usual" defined as follows: usual behavior for alerts generation will be defined using the first 6 weeks of the study execution. Then a rolling window of 6 weeks will be moved every 2 weeks to recalculate the new 'usual' activities with the 'new' last 6 weeks. The rolling window will not include in the computation of the new behavior 2 weeks periods for which a decrease of the activity has been identified (generating an alert) to avoid underestimating the usual behavior.*

| Adverse Event | User examples | Tips and recommendations |
|---|---|---|
| **Fatigue** | I'm tired all the time<br>I am always tired<br>I feel tired<br>I don't have any energy<br>I feel knackered<br>I'm shattered<br>I feel exhausted<br>I am limited in everyday activities<br>I need to rest often<br>I often need to stop my activities<br>I feel less strong<br>I have no energy | Fatigue is a common side effect of cancer treatments. There is no treatment for fatigue, but extra rest can help. If it gets worse and stops you doing everyday activities, please let your clinician know.<br>Here are some helpful tips to help you with feeling fatigued:<br>● Make a plan that balances rest and activity<br>● Plan time to rest when you are very tired<br>● Try to keep a regular sleep routine.<br>● Eat well<br>● Keep active<br>Other tips may be found at URL:<br>https://www.cancer.gov/about-cancer/treatment/side-effects/fatigue<br><br>And<br><br>https://www.nhs.uk/live-well/sleep-and-tiredness/<br><br><br>If your feeling of fatigue lasts longer than a week, speak to your GP.<br><br>Is there any other issue (e.g. pain) you have suffered from in the last weeks? If yes, which one? |
| **Fever** | I have a fever<br>My forehead is very hot<br>I have chills<br>My skin feels clammy<br>I feel feverish<br>I have a temperature<br>I feel very hot | You may have a high temperature if:<br>● your chest or back feel hotter than usual<br>● you have shivering (chills), sweating or warm, red skin<br>● your temperature is 38C or above<br><br>A high temperature (fever) is often caused by an infection.<br>● Get plenty of rest<br>● Drink plenty of fluid (if there is no medical contraindication)<br>● Ask your GP if you may take any medication<br>● Don't forget to wash your hands after going to the bathroom and before preparing food<br>● Contact your health care team if you have signs of an infection.<br>More tips can be found at URL:<br>https://www.cancer.gov/about-cancer/treatment/side-effects/infection<br><br><br>A high temperature can be a sign of coronavirus. To get more advice on this please go to: https://www.nhs.uk/conditions/coronavirus-covid-19/symptoms/main-symptoms/<br><br>Did your fever come out together with other issues? |

| | | |
|---|---|---|
| **Malaise** | I feel out of sorts<br>I feel uneasy<br>I feel unwell<br>I feel uncomfortable<br>I have malaise<br>I don't feel well | Malaise is a sensation of general discomfort. It might be due to other underlying symptoms such as fever or fatigue. Please, provide further details about your symptoms.<br><br>Is your malaise associated with fatigue? [If yes, then show tips about fatigue] |
| **Excessive sleepiness** | I feel sleepy all the time<br>(could relate to fatigue)<br>I want to get back to bed<br>I feel drowsy<br>During the day I feel the need to sleep<br>I'm always sleepy | Try to go to bed only when sleepy<br>● Try to sleep in a quiet and dark room, and in a comfortable bed.<br>● Avoid alcohol consumption before sleeping.<br>● Try to wake up and go to bed at regular hours.<br><br>Does your excessive sleepiness follow a difficulty in sleeping? [If yes, then show tips about difficulty sleeping]<br><br>Have you been prescribed new drugs? If so, ask your GP whether they might induce excessive sleepiness among the anticipated adverse events. |
| **Difficulty sleeping** | I can't sleep<br>I wake up at night all the time<br>I have insomnia<br>I am sleepless<br>I spend my nights awake<br>I can't get to sleep | Difficulty sleeping is very common.<br><br>● Try to stop watching television or using electrical devices a couple of hours before going to bed.<br>● Avoid drinking or eating a lot before bedtime.<br>● Avoid exercising a few hours before bedtime.<br>● Avoid drinking alcohol before bedtime.<br>● Try to sleep in a quiet and dark room, and in a comfortable bed<br>● Try to wake up and go to bed at regular hours<br><br>Is your difficulty in sleeping related to any other issue? Are you anxious, depressed or do you suffer from any new symptom you were not aware of before? |

| | | |
|---|---|---|
| **Negative emotions / Depression** | I feel down all the time<br>My life is terrible<br>Nothing makes sense anymore<br>I feel no joy in doing anything<br>I have less interest or pleasure in my activities<br>I feel depressed<br>I noticed a slowing down of thought<br>I feel worthless<br>I feel excessively or inappropriately guilty<br>I have less ability to think or concentrate<br>I am more indecisive than before<br>Sometimes I would prefer to die<br>I often think about my death<br>I feel alone<br>I always feel guilty | We all feel sad at times. Remember that you're not alone, feeling down is a common problem and help is available<br>Here are some helpful tips to help you when feeling down:<br>● Try talking about your feelings to a friend, family member, your doctor or a counsellor<br>● Taking more exercise, cutting down on alcohol and eating healthily can help.<br>● Do things that you enjoy<br>● Try setting yourself some small easy to achieve targets each day<br>● Express your feelings by talking to friends or family.<br>● Try to stay positive by focusing on what you can do to stay as healthy as possible<br>● Don't blame yourself for your cancer<br>For more suggestions you can also have a look here:<br>https://www.cancer.gov/about-cancer/coping/feelings/depression-pdq#_49<br>and:<br>https://www.nhs.uk/conditions/stress-anxiety-depression/low-mood-and-depression/<br><br>Do you think that your sadness could be related to any new symptom (e.g. pain or difficulty in swallowing)? |
| **Change in social circumstances** | I lost my job<br>I lost my home<br>My wife left me<br>My husband left me<br>My wife left me<br>My husband left me<br>My partner left me<br>My girlfriend left me<br>My boyfriend left me<br>I left my wife<br>I left my husband<br>I left my partner<br>I left my girlfriend<br>I left my boyfriend<br>Found a job<br>Met someone | Cancer is often associated with many social and practical changes, such as problems with finances, employment, legal matters, relationships. Here are some tips to help you cope with these changes:<br>● Talk about with friends or family.<br>● Let family and friends help you.<br>● Expect relationships to change.<br>● Stay involved in social activities.<br>For more suggestions you can also have a look at:<br>https://www.cancer.net/coping-with-cancer/talking-with-family-and-friends/how-cancer-affects-family-life<br><br>Did your changes in social circumstances occur together or after a period in which you have had any new symptom? |

| | | |
|---|---|---|
| **Neck swelling** | I've got a swollen neck<br>My neck is swollen<br>I have a lump on my neck<br>My neck feels bloated<br>Some parts of my body are swelling – I might have OEDEMA, what should I do? | Cancer treatments can cause changes in your neck and sometimes these changes can happen much later after your treatment<br>Neck swelling can be caused by oedema secondary to chemotherapy, radiotherapy and surgical procedures<br><br>If it is painful you can take painkillers such as paracetamol or ibuprofen (only if your GP agrees, if you are able to and if you are not allergic to these drugs; please ask your doctors for the correct and maximum dosage)<br>Chat to your head and neck cancer specialist nurse or your doctor about the swelling<br><br>Some tips to prevent or lessen the neck swelling:<br>● Try gentle neck stretches: gently turn your head from side to side several times. Also try gently dropping your chin towards your chest. A physiotherapist can advise you on specific exercises.<br>● Try a gentle massage of the area with the help of moisturizing cream. A physiotherapist can show you how to perform specific massages.<br>● Avoid clothes with a high or narrow collar<br><br>If you have found a new lump in your neck please contact your GP or tell your head and neck cancer team.<br><br>Is your neck oedema associated with breathing difficulties? [in this case, ask for specific questions about difficulty breathing] |
| **Facial pain** | My face hurts<br>I have pain on my face<br>I feel pain on my face/cheek/mouth/lips/forehead/brow<br>I have an ache on my face/cheek/mouth/lips/forehead/brow<br>I need to take pain killers for my face/cheek/mouth/lips/forehead/brow<br>My face is sore | Pain can be a side effect of cancer treatments but can be controlled so that it doesn't interfere with your daily life.<br><br>Some tips:<br>● Keep a note of when you get pain and what makes the pain worse<br>● Ask your GP whether you can take a pain killer. In case, don't forget to take any prescribed pain medication<br><br>● If you still have pain after using pain killers, please contact your doctor. They can refer you to a pain specialist if needed. |

| | | |
|---|---|---|
| **Difficulty breathing** | I'm having trouble breathing<br>Can't breathe<br>I'm experiencing shortness of breath<br>My nose feels blocked<br>Can't breathe through my nose<br>I feel my airways are closed<br>I feel like I am suffocating<br>I feel like something smothers/chokes/stifles me<br>I am short of breath | Shortness of breath can be caused by lots of different things. Common causes include a cold or a chest infection. Treatment will depend on the cause of your symptoms.<br><br>If you are suddenly struggling to breathe and your chest feels tight and / or you have pain that spreads to your neck, jaw, arms and back then you do need to see a doctor urgently. Please call 999.<br><br>If you experience shortness of breath and have already seen a doctor:<br>● Try doing breathing exercises. Go to How can I manage my breathlessness? \| British Lung Foundation (blf.org.uk) for advice<br>● Don't overdo exercise.<br>● Take periods of rest during the day.<br>● If your breathing difficulty is new and persistent, please contact your doctor.<br><br>Follow the advice given at:<br>https://www.nhs.uk/conditions/shortness-of-breath/<br><br>If your nose always feels blocked:<br>● Try saline drops or sprays or rinse with a salt water solution.<br>● Try decongestant sprays.<br>● Sleep with your head elevated.<br><br>If it does not resolve, go to the emergency room [high priority alert]. |
| **Nose bleeds** | My nose is bleeding<br>My nose is always bleeding<br>I get nose bleeds<br>Last night I was bleeding from my nose<br>I found some blood after blowing my nose<br>I found some blood in my nose mucus<br>I had epistaxis | Nosebleeds are common and can usually be easily treated at home. When you get a nosebleed:<br>● Sit or stand, lean forward and breathe through your mouth. Pinch your nose just above your nostrils for around 10 minutes<br>● You can try putting some ice (wrapped in a cloth) at the top of your nose<br>● Avoid medicines that can increase your risk of bleeding, such as aspirin or ibuprofen. Check with your doctor.<br><br>After a nosebleed for the next 24 hours:<br>● If you need to blow your nose do it very gently.<br>● Avoid hot drinks.<br>● Try not to do heavy lifting or vigorous exercise.<br>● Sleep with your head elevated.<br><br>Contact your doctor if you get regular nosebleeds or if it lasts more than 15 minutes.<br><br>Follow the advice at: https://www.nhs.uk/conditions/nosebleed/<br><br>If it is very heavy and it does not resolve, go to the emergency room [high priority alert]. |

| | | |
|---|---|---|
| **Difficulty speaking** | I'm having trouble communicating<br>Can't speak to anybody<br>My throat feels blocked<br>I find it difficult to speak<br>I cannot say words properly<br>I cannot talk like before<br>My throat hurts when I speak | If you were operated on for cancer in the oral cavity, it is likely that you may have to live with your difficulty in speaking. If this symptom is worsening, please chat to your doctor, they may refer you to a speech therapist for advice.<br><br>If your throat is sore try gargling with warm salty water. Drink plenty of fluids (not too hot, and only if there is no medical contraindication). If you smoke try to cut down.<br><br>If a sore throat or a feeling of a lump in your throat persists please speak to your doctor.<br><br>Is your difficulty in speaking associated with troubles in eating or swallowing? [if yes, then show the specific tips] OR [No, I can swallow without problems.] |
| **Dry mouth** | I'm feel thirsty all the time<br>Feels like my mouth is really dry<br>I need to drink often<br>I have xerostomia<br>I wake in night with a dry mouth<br>I have no/less saliva<br>My mouth is arid | Treatments for head and neck cancer often affect saliva production But a dry mouth can also be caused by dehydration , or eating lots of salty foods. Some medication can also cause your mouth to feel dry<br>● Keep a bottle of water with you and sip regularly<br>● If you feel thirsty it may be that you are not drinking enough fluids. Aim for 6 – 8 glasses of fluid each day (only if there is no medical contraindication).<br>● Try using chewing gum to increase saliva.<br>● If you are already drinking plenty of fluid and you still feel thirsty please contact your doctor<br><br>You may want to try some products that are available from online shopping websites. These products will not cure your dry mouth, but some people who have received similar treatments to yours have found them helpful. |
| **Toothache / Lost a tooth** | I lost a tooth<br>My tooth fell out<br>My tooth hurts<br>My teeth hurt<br>My gums hurt<br>My gum hurts<br>My gums bleed<br>Bleeding gums | Cancer treatments can sometimes cause dental problems.<br>● Check your mouth every day<br>● Gently brush your teeth, gums and tongue with a soft brush after each meal and before going to bed at night<br>● Rinse your mouth daily with an alcohol free mouthwash<br>● Book a check-up with your dentist / dental hygienist every six months |
| **Muscle weakness** | I'm feeling weak<br>Can't lift anything<br>Can't use the stairs<br>I have difficulty in doing exercise<br>I have joint pain- what should I do?<br>[to be differentiated from fatigue] → Is your weakness a generalized symptom (fatigue) or do you have also muscle pain | - Use the same recommendations used for fatigue -<br><br>Is your muscle weakness associated with generalized fatigue? [if yes, then ask the same questions used for fatigue] |

| | | |
|---|---|---|
| **Ear pain** | My ear hurts<br>Outside of my ear hurts<br>My ear hurts inside<br>I have earache<br>I have otalgia<br>I have pain in my ear | Pain in one or both ears can occur for many reasons, some not related to the ear at all.<br><br>When the pain is caused by an ear problem, the most common reasons are: blockage of the passageway between the middle ear and the back of the throat (the Eustachian tube); ear infection. Pain from a sore throat or a problem with the jaw joints (called temporomandibular joint disorders - TMJ) may be felt in the ear.<br>Some tips to help with ear pain:<br>● Ask your GP if you can take painkillers<br>● Please don't put anything, including cotton buds, inside your ear.<br>● Try resting the infected ear on a warm (not hot) heating pad<br>● Try neck exercises and neck massage<br>● Eat liquid / soft foods<br><br>If the pain persists, contact your GP. Your pharmacist can also help give advice.<br><br>Is your ear pain associated with difficulty in swallowing? [if yes, then show the specific tips about difficult swallowing] |
| **Difficulty hearing** | Can't hear when people are talking<br>I can't hear the TV<br>I hear constant buzzing in my ears<br>I often need to turn up the volume<br>I often ask people to speak louder<br>I ask people to lift their voice<br>I cannot understand people talking with soft voice<br>I am hard of hearing<br>People speak too quietly | There are lots of different reasons why people can suffer from hearing loss.<br><br>People treated with cisplatin or radiotherapy to the nasopharynx may suffer from hearing loss.<br><br>See your GP so that they can look into why you are having problems with hearing. |
| Tinnitus | I hear noise in my ears<br><br>I hear ringing, buzzing, roaring, clicking. | If you hear a constant ringing in your ears or in your head, this condition is called tinnitus.<br>There are many reasons why people treated for head and neck cancer can suffer from tinnitus. Often this symptom is associated with hearing loss.<br><br>Tinnitus may be temporary or, sometimes, a chronic condition. In any case, try to listen to some soft music. On the internet there are many relaxing videos with a soft musical background or with a simil-natural background (e.g., hearing the sea waves or countryside sounds etc). These videos or audios will not resolve your problem, but you may benefit from them because the tinnitus may be less obvious or intrusive.<br><br>Do you feel more difficulty in hearing than before?<br>[if yes, then go to difficulty hearing section] |

| | | |
|---|---|---|
| **Vertigo** | The world seems to revolve<br>I have vertigo<br>I feel dizzy<br>I have dizziness<br><br>I feel nauseous while moving<br>I feel like vomiting while moving<br>I feel travel sickness | Vertigo is when you feel that everything around you is spinning and your balance is affected<br>● Try lying down in a quiet room to help stop the feeling of spinning<br>● If you feel dizzy, sit or lie down and when you get up, get up slowly.<br>● Try to relax, feeling anxious can make vertigo worse |
| Nausea | I feel like I have to vomit<br>I feel nauseated<br>I feel queasiness<br><br>[try to intercept any word including vomit, vomiting, or nausea] | If you feel nausea:<br>● ask your GP whether you may try an over-the-counter anti-nausea medicine.<br>● Avoid greasy, fried, sweet, or spicy foods if you feel sick after eating them.<br>● Try cold foods that do not have strong smells, or let food cool down before you eat it.<br>● Try foods or drinks containing ginger, some people find this helps<br>● Try eating smaller meals , it may help to eat less but more often<br><br>Is your nausea related to the introduction of new drugs? If yes, please contact your GP: nausea may be a drug-related adverse event for many medicines.<br><br>Is your nausea related with body movements (e.g. do you feel dizzy)? [if yes, then go to vertigo tips]<br><br>Is your nausea associated with any other further symptom? [if yes, then go to the specific tips] |
| **Diarrhea** | I have had diarrhoea for the last two days – what should I do.<br>I have loose stools<br>My stools/faeces are watery<br>I go to the toilet more often than usual<br><br>[avoid letting the user saying swear words -> do no recognize them] | When you have diarrhoea it is important to<br>● Drink plenty of fluid (only if there is no medical contraindication).<br>● Eat small meals that are easy on your stomach. Eat six to eight small meals throughout the day, instead of three large meals.<br>● Wash your hands frequently<br>● Phone your pharmacist or general practitioner for advice on medication that can be taken.<br>● Try to keep your anal area clean and dry.<br>●<br><br>Is your diarrhea related to the introduction of new drugs? If yes, please contact your GP: diarrhoea may be a drug-related adverse event for many medicines.<br><br>Is your diarrhoea associated with any other further symptom? [if yes, then go to the specific tips] |

| | | |
|---|---|---|
| **Constipation** | I'm having trouble in the bathroom<br>I'm constipated, what can I do?<br>I have hard stools<br>I go to the toilet less frequently than usual<br>My stools/poo are often large and dry, hard or lumpy<br>I am straining or in pain when I go to the bathroom/have a poo | Constipation is very common and can affect people of all ages. It can usually be treated at home with simple changes to your diet and lifestyle<br><br>● Eat high-fibre foods.<br>● Drink plenty of liquid (only if there is no medical contraindication).<br>● Try to be active every day.<br>● If it persists talk to your local pharmacist about medication that can help<br><br>Is your constipation related to the introduction of new drugs? If yes, please contact your GP: constipation may be a drug-related adverse event for many medicines.<br><br>Is your constipation associated with any other further symptom? [if yes, then go to the specific tips] |
| **Difficulty seeing** | I'm having trouble seeing<br>My eyes don't work as well as they used to<br>The light is too bright<br>I can't see as well as I used to<br>My vision has decreased<br>I see strange flashing lights<br>Having trouble seeing at night | Treatments for head and neck cancer might affect your eyes but there are many other reasons for reduced vision.<br><br>An optician can test your eyesight and see if you need glasses or an opthalmologic consultation.<br><br>Is your difficulty in seeing associated with headache? [if yes, go to pain tips]<br><br>Is your difficulty in seeing associated with any other further symptom? [if yes, then go to the specific tips]<br><br>Is your difficulty in seeing a total blindness? In this case, go to the emergency room [high priority alert] |
| **Dry eye** | My eyes are so dry these days<br>I feel as if I have sand in my eyes<br>I have xerophthalmia<br>My eyes are arid<br>Feels like grit in my eye | Many people suffer with dry eyes.<br><br>● Try to take regular breaks from looking at the TV or computer screens<br>● Try putting a warm compress over your eyes for five minutes, use a clean cloth rinsed in hot water<br>● Try moisturising eye drops, you can get these from your local chemist.<br><br>Is your dry eye associated with difficulty in seeing? [if yes, go to tips about difficulty in seeing]<br><br>Is your dry eye associated with any other further symptom? [if yes, then go to the specific tips] |
| **Eye pain** | My eyes hurt<br>My eye hurts<br>I have pain in my eyes<br>Sore eyes | There are lots of reasons for sore eyes<br>● Avoid rubbing your eyes<br>● Drink plenty of water (only if there is no medical contraindication) and get plenty of rest<br>● If you think you have an infection, or the pain does not go away, contact your GP<br><br>Is your eye pain associated with difficulty in seeing? [if yes, go to tips about difficulty in seeing] |

| | | |
|---|---|---|
| | | Is your dry eye associated with any other further symptom? [if yes, then go to the specific tips]<br><br>Is your eye pain associated with total blindness? In this case, go to the emergency room [high priority alert] |
| **Nervous eyelid** | My eye twitches<br>I have nervous eyelids | Eye twitches are common and usually stop on their own.<br><br>Sometimes eye twitching can be a late complication of cancer treatments so if they last longer than two weeks please see your doctor.<br><br>Please provide me with further information about any further symptoms that you have. |
| **Eye floaters** | I see little floaters in front of me<br>I see little flies/worms/insects in my eyes<br>It's like there were flies/worms/insects in my eyes<br>I see dark shapes in my eye | Floaters are small dark shapes that float across your vision. Almost everyone develops floaters as they get older, they come and go and in most cases do not require treatment.<br><br>If a lot of new floaters appear suddenly and don't go away it's important to tell your doctor<br><br>● Use a saline solution to rinse your eyes, if there's discharge<br>● If you need to rub your eyes, rub your eyes very gently<br>Are your eye floaters associated with difficulty in seeing? [if yes, go to tips about difficulty in seeing]<br><br>Are your eye floaters associated with dry eye? [if yes, go to tips about dry eye]<br><br>Is your dry eye associated with any other further symptom? [if yes, then go to the specific tips]<br><br>Are your eye floaters associated with total blindness? In this case, go to the emergency room [high priority alert] |
| **Swollen eye** | My eye is swollen<br>My face is swollen around my eyes<br>My eyes feel bloated<br>I have oedema around my eye<br>My eye looks like someone punched me in the face | There are many reasons for swelling on the eyelid or around the eye.<br><br>● Try a saline solution to rinse your eyes, if there's discharge.<br>● Try a cool compress over your eyes. This can be a cold clean washcloth.<br>● Remove contacts, if you have them.<br>● Elevate your head at night to reduce fluid retention.<br>● See your doctor if the problem persists<br><br>Is your swollen eye associated with difficulty in seeing? [if yes, go to tips about difficulty in seeing]<br><br>Is your swollen eye associated with dry eye? [if yes, go to |

| | | |
|---|---|---|
| | | tips about dry eye]<br><br>Is your swollen eye associated with eye pain? [if yes, go to tips about eye pain]<br><br>Does your eye bleed? [if yes, then go to tips about bleeding eyes]<br><br>Is your dry eye associated with any other further symptom? [if yes, then go to the specific tips]<br><br>Is your swollen eye associated with total blindness? In this case, go to the emergency room [high priority alert] |
| **Bleeding eyes** | My eyes bleed<br>There's blood in my tears | Is your bleeding light or heavy?<br><br>[if heavy, then write the following tips]<br><br>If the bleeding is very heavy and it does not resolve, go to the emergency room [high priority alert].<br><br>Is your bleeding eye associated with total blindness? In this case, go to the emergency room [high priority alert]<br><br>[if light, then write the following tips]<br>Sometimes a blood vessel in the eye can burst causing your eye to look very red. This will usually settle but if it does not go away see your doctor.<br>If your eye is bleeding this can be due to several causes, see your doctor who can find out what is causing this to happen.<br>● If you need to rub your eyes, rub your eyes very gently.<br>● Try a saline solution to rinse your eyes<br>● Ask your GP to check if you can avoid medicines that can increase your risk of bleeding, such as antinflammatory agents or anticoagulants. To be sure check with your doctor<br>● Try using a cool compress over your eyes<br><br>Is your bleeding associated with difficulty in seeing? [if yes, go to tips about difficulty in seeing]<br><br>Is your bleeding associated with swollen eye? [if yes, go to tips about swollen eye]<br><br>Is your bleeding eye associated with nosebleed? [if yes, go to tips about nose bleeding]<br><br>Is your bleeding eye associated with eye pain? [if yes, go to tips about eye pain]<br><br>Is your dry eye associated with any other further symptom? [if yes, then go to the specific tips]<br><br>If the bleeding is very heavy and it does not resolve, go to the emergency room [high priority alert]. |

| | | |
|---|---|---|
| | | Is your bleeding eye associated with total blindness? In this case, go to the emergency room [high priority alert] |
| **Eye watering** | My eyes keep watering<br>I feel like I'm crying but I'm not sad<br>I have a lot of tears<br>My eyes are streaming but I'm not crying | Watery eyes are common and often get better without treatment. It can be normal in cold weather or if you have an allergy.<br><br>● If you need to rub your eyes, rub your eyes very gently<br>● try a saline solution to rinse your eyes.<br>● See your doctor if you have lumps or swelling around the eye or if eye watering is stopping you from doing your daily activities.<br><br>Is your eye watering associated with any other further symptom? [if yes, then go to the specific tips] |
| **Sexuality Issues** | I'm having issues with sexuality<br>I'm having trouble downstairs<br>I feel less/more interest in sex<br>I am impotent<br>I have less libido<br>I've been having less sex with my partner<br><br>[avoid letting the user saying swear words -> do no recognize them] | Many types of cancer and treatments for cancer can cause sexual side effects, such as loss of desire for sexual activity or inability to have an orgasm.<br>Loss of sex drive and / or problems with sexual function are very common<br>Don't be afraid to chat to your GP, they are used to talking about this<br><br>Here are some helpful tips:<br>● Talk to your partner and share your feelings.<br>● Learn ways to adapt to changes in sexual function and to be intimate.<br>● Let your doctor know if you are having problems with intimacy or sex.<br><br>For more suggestions you can also have a look here:<br>https://www.ncbi.nlm.nih.gov/books/NBK65919/<br>https://www.cancer.gov/about-cancer/coping/self-image<br>● Or try<br>https://sexualadviceassociation.co.uk/<br><br>Are your sexuality issues related to sadness? [if yes, go to depression]<br><br>Are your sexuality issues associated with any other further symptom? [if yes, then go to the specific tips] |
| **Weight loss** | I lost weight<br>I weigh less than before<br>How did I lose so much weight? | Losing weight when you don't mean to can happen as a result of stress, but it can also be a sign of illness. If you keep losing weight and you are not on a diet please see your |

| | | |
|---|---|---|
| | I cannot recover to my prior weight<br>I am not on a diet, but I have lost weight<br>I have lost a lot of weight<br>Eating lots but losing weight<br>Can't get fatter | doctor.<br><br>● Drink plenty of fluids (if your GP agrees).<br>● Choose healthy but high calorie foods.<br>● Doing some physical activity might help you to increase your appetite.<br>● Please, specify if you have troubles with swallowing.<br><br>Was your weight loss intentional? If not, was it associated with sadness? [if yes, go to depression]<br>Was it associated with difficulty in swallowing? [if yes, go to difficulty swallowing]<br><br>Is your weight loss associated with any other further symptom? [if yes, then go to the specific tips] |
| **Difficulty swallowing** | I can't eat* [follow-up]<br>Food doesn't go down my throat<br>I can't swallow food<br>I feel like there is a lump in my throat when swallowing<br>I have dysphagia<br>I can only swallow very soft food<br>Trouble eating solid food | If this is a new problem please let your doctor know.<br><br>If you have difficulty in swallowing solid foods, choose soft foods (e.g. pureed or mashed).<br><br>Please, specify if you have had weight loss or if you have a cough after swallowing.<br><br>Did you have some weight loss? [if yes, then go to weight loss]<br><br>Do you usually cough after swallowing? [if yes, then tell the participant to ask for a medical consultation]<br><br>Your doctor may refer you to see a dietitian and a speech and language therapist.<br><br>While waiting for your specialist or GP consultation, remember to avoid drinking a lot of liquids in the same occasion, preferring modest quantities of fluids. While swallowing, be concentrated on what you are doing, and try to keep your head down.<br><br>Is your difficulty in swallowing associated associated with fever? [if yes, go to fever, and tell the participant to ask the doctor] |
| **Mouth sores** | Can't eat* [follow-up]<br>My mouth is full of sores<br>I have mouth sores<br>My mouth is painful<br>I have some red/painful/fluid-filled blisters that appeared near the mouth and lips<br>I noticed some ulcers in my mouth/on my lips<br>I have some aphthae in my mouth/on my lips<br>I have mouth ulcers<br>I have an ulcer on my lip<br>I have an ulcer on my tongue<br>Painful tongue | Mouth ulcers are common and should heal in a week or so.<br><br>● Avoid smoking or using tobacco products.<br>● Try to drink plenty of fluids (only if there is no medical contraindication).<br>● Choose foods that are soft, and easy to swallow.<br>● Avoid crunchy foods like toast<br>● Avoid very spicy or salty foods<br>● Soften dry foods with gravy, sauce, or other liquids.<br>● Try a soft bristle toothbrush<br>● Your local pharmacist can advise on treatments to reduce the pain<br>● Eat a healthy balanced diet<br>● See your doctor or dentist if the ulcers keep |

| | | |
|---|---|---|
| | | coming back or if they haven't healed after 3 weeks<br>Please, specify if you have had weight loss or if you have cough after swallowing (aspiration).<br><br>While waiting for your specialist or GP consultation, keep your mouth clean and have daily washes with water and sodium bicarbonate. |
| **Appetite loss** | I don't feel like eating<br>I don't have an appetite<br>I have lost my appetite<br>I don't want to eat | Loss of appetite can happen for many reasons.<br><br>Treatments like radiotherapy or chemotherapy can affect your appetite.<br>Remember to drink plenty of fluids (only if there is no medical contraindication).<br>Try eating little and often<br>For more on appetite loss you can read:<br>https://www.cancer.gov/about-cancer/treatment/side-effects/appetite-loss<br><br>Did your appetite loss make you lose weight? [if yes, go to weight loss tips]<br><br>Was your appetite loss associated with sadness? [if yes, go to depression]<br>Was it associated with difficulty in swallowing? [if yes, go to difficulty swallowing]<br>Was it associated with difficulty in opening your mouth? [if yes, go to difficulty opening mouth] |
| **Difficulty opening mouth** | I'm struggling to open my mouth<br>My mouth won't open<br>I can't eat*<br>I have trismus | Not being able to open your mouth (trismus) fully can happen after oral surgery or can be the result of other problems.<br><br>● Try opening and closing your mouth regularly to work the muscles.<br>● You may need to change to softer foods until your symptoms improve<br>● If this symptom persists, please tell your doctor, they may refer you to a speech therapist for advice. |

| | | |
|---|---|---|
| **Difficulty eating** | I can't eat*<br>I eat less than usual<br>I only eat soup<br>I can only drink liquids<br>I hardly ever eat anything<br>Why is eating so hard?<br><br>I'm having trouble eating<br>Difficulty eating<br>Struggling with eating<br>I only eat soup<br>I eat less than usual<br>I only drink liquids<br><br>(to be differentiated from difficulty in swallowing) → follow-up question: Is your difficulty in eating more related to swallowing or to chewing? | What do you mean by difficulty in eating?<br><br>Have you had any difficulty in swallowing? [if yes, go to difficulty swallowing]<br><br>Did your difficulty eating make you lose weight? [if yes, go to weight loss tips]<br><br>Was this symptom associated with sadness? [if yes, go to depression]<br><br>Was it associated with mouth sores?[if yes go to tips about mouth sores]<br><br>If you have been operated on for oral cavity cancer, it might take time to recover from difficulties in eating.<br>If this symptom is associated with swallowing problems, please contact your doctor who can investigate further.<br>If this symptom is not associated with swallowing problems, please tell your doctor, they may refer you to other specialists. |
| **Increased sensitivity to smells** | Smells bother me<br>Everything stinks<br>I can smell everything<br>Things smell different<br>Everything smells weird | There are many reasons for an increase in sensitivity to smells. It will normally resolve by itself.<br><br>• Try eating foods that don't have a strong flavour.<br>• Avoid using perfumes and scented products<br><br>Is your increased sensitivity to smells associated with nausea? [if yes, then provide tips about nausea] |
| **No taste** | I can't taste food<br>Food doesn't taste the same<br>I keep adding salt, but nothing is working<br>I have dysgeusia<br>Water tastes like it is salted<br>Every food seems bitter/disgusting/with no taste/tasteless to me<br>Everything lacks in flavour | Loss of taste can occur for many reasons, most are short term but if it persists please see your doctor.<br><br>Treatments such as radiotherapy can sometimes cause a temporary loss of taste. If this persists, contact your doctor for further details.<br><br>Loss of taste can be a symptom of Covid-19, if this is a new symptom please take a PCR test as soon as possible. See Get a free PCR test to check if you have coronavirus (COVID-19) - GOV.UK (www.gov.uk)<br><br>If you do not have mouth sores, try adding plenty of spices and herbs to your food to increase flavour.<br><br>• Be aware - if you have lost your sense of taste you may not notice when food has gone off. Take a look at the advice offered on the Fifth Sense website Safety Advice – Fifth Sense<br><br>Is your loss of taste associated with a loss of smell? [if yes, then go to tips about loss of smell] |

| | | |
|---|---|---|
| **Loss of smell** | I can't smell anything<br>Loss of smell<br>Flowers don't smell any more | Changes in your sense of smell are often caused by a cold or an allergy.<br>Treatments for head and neck cancers can also affect your sense of smell, in this case it might take time to recover from loss of smell.<br>● A loss of smell can be a symptom of Covid-19, if this is a new symptom please take a PCR test as soon as possible. See Get a free PCR test to check if you have coronavirus (COVID-19) - GOV.UK (www.gov.uk)<br>● Try rinsing inside your nose with a salt water solution (you can make this or buy ready made)<br>● Be aware - if you have lost your sense of smell you may not notice things like gone off food or a gas leak. Take a look at the advice offered on the Fifth Sense website Safety Advice – Fifth Sense<br><br>If you have been treated for nasopharyngeal cancer, remember to do your nasal washes daily. |

Supplementary Table 2. Chatbot utterances

| Domain | Activity | Detected / Derived through | Storage frequency | Data format | Mobile applicatio n |
|---|---|---|---|---|---|
| **Mobility** | **Active** | Google Fit API- step counter | Once a day | Number of steps / day / hour | B1.1 & B3.1 |
| | **Inactive** | Activity Transition Recognition API | 1 minute | Activity type, timestamp | B3.2 |
| **Physical activity** | **Walking** | Activity Transition Recognition API | 1 minute | Activity type, timestamp | B3.2 |
| | **Any fitness activity** | Activity Transition Recognition API | 1 minute | Activity type, timestamp | B3.2 |
| | **Sports** | GPS Location API and correlation with Open Street places and Foursquare (for detecting location within a sports centre), and Activity Recognition API | 1 minute for GPS location and 1 minute for Activities | GPS (Lat, Lng), Activity type, timestamp | B1.1 & B3.2 |
| **Non Sleep Events Ratio (NSER)** | **Not sleeping (awake time ratio) during the night** | Screen ON status from mobile device divided by the total time one is lightly to sleep | 1 minute (during probable sleep time) | Screen status & timestamp / total time (in minutes) one is likely to sleep | A3 |
| **Activities of Daily Living** | **Eating/drinkin g** | GPS Location API and correlation with Open Street places and Foursquare (for detecting places for eating or drinking) | 1 minute for GPS location and once a day for POIs | GPS (Lat, Lng), POI information | B1.1 |
| | **Staying at home** | GPS Location API | 1 minute for GPS location and once a day for POIs | GPS (Lat, Lng), POI information | B1.1 |
| **Instrumental Activities of Daily Living** | **Shopping** | GPS Location API and correlation with Open Street places and Foursquare (for detecting places shopping) | 1 minute for GPS location and once a day for POIs | GPS (Lat, Lng), POI information | B1.1 |
| | **Manage medications** | GPS Location API and correlation with Open Street places and Foursquare (for detecting health related places) | 1 minute for GPS location and once a day for POIs | GPS (Lat, Lng), POI information | B1.1 |
| | **Travelling** | GPS Location API and correlation with | 1 minute for GPS location and once a | GPS (Lat, Lng), POI | B1.1 |

| | | | | | |
|---|---|---|---|---|---|
| | | Open Street places and Foursquare (for detecting travelling related places) | day for POIs | information | |
| | **Managing finances** | GPS Location API and correlation with Open Street places and Foursquare (for detecting finances related places) | 1 minute for GPS location and once a day for POIs | GPS (Lat, Lng), POI information | B1.1 |
| **Socialization** | **Visiting social places** | GPS Location API and correlation with Open Street places and Foursquare (for detecting social places for eating, drinking, music, theatre, etc.) and number of wifi connections | 1 minute for GPS location and once a day for POIs | GPS (Lat, Lng), POI information | B1.1 & B1.2 |
| | **Phone Usage** | Daily sum of duration in calls + total duration of all applications used in device | Once a day | Duration in minutes | B2.1 & B2.3 |
| | **Using social networks** | Detailed (duration) daily analysis of user using social media networks from the phone (whatsapp, messenger, linkedin, zoom, skype, viber, telegram, etc) | Once a day | Application name, duration per day (minutes) | B2.3 |
| **Cognitive function** | **Visiting cultural places** | GPS Location API and correlation with Open Street places and Foursquare (for detecting places such as cinemas, museums, etc.) | 1 minute for GPS location and once a day for POIs | GPS (Lat, Lng), POI information | B1.1 |
| | **Self-care** | GPS Location API and correlation with Open Street places and Foursquare (for detecting places such as spa, gyms, hairdresser, etc.) | 1 minute for GPS location and once a day for POIs | GPS (Lat, Lng), POI information | B1.1 |
| | **Spirituality** | GPS Location API and correlation with Open Street places | 1 minute for GPS location and once a day for POIs | GPS (Lat, Lng), POI information | B1.1 |

| | | | | | |
|---|---|---|---|---|---|
| | | and Foursquare (for detecting places such as church, synagogue, mosque, etc.) | | | |
| | **Education** | GPS Location API and correlation with Open Street places and Foursquare (for detecting places such as school, university, etc.) | 1 minute for GPS location and once a day for POIs | GPS (Lat, Lng), POI information | B1.1 |
| **Affections** | **Sentiment** | Textual exchanges with WP5 chatbot app | Real time | Affection type, timestamp | *Chatbot* |
| | **Depression** | Textual exchanges with WP5 chatbot app | Real time | Affection type, timestamp | *Chatbot* |
| **Health related** | **Visit heath related places** | GPS Location API and correlation with Open Street places and Foursquare (for detecting places such as GP practice, hospital, etc.) | 1 minute for GPS location and once a day for POIs | GPS (Lat, Lng), POI information | B1.1 |

Supplementary Table 3. Domains and activities as collected by the study App
